# Targeting the β2-integrin LFA-1, reduces adverse neuroimmune actions in neuropathic susceptibility caused by prenatal alcohol exposure

**DOI:** 10.1186/s40478-019-0701-y

**Published:** 2019-04-08

**Authors:** Joshua J. Sanchez, Jacob E. Sanchez, Shahani Noor, Chaselyn D. Ruffaner-Hanson, Suzy Davies, Carston R. Wagner, Lauren L. Jantzie, Nikolaos Mellios, Daniel D. Savage, Erin D. Milligan

**Affiliations:** 1 Department of Neurosciences, School of Medicine, University of New Mexico Health Sciences Center, MSC08 4740, Albuquerque, NM 87131-0001 USA; 2 Department of Anesthesiology and Critical Care Medicine, University of New Mexico Health Sciences Center, MSC08 4740, Albuquerque, NM 87131-0001 USA; 3 Department of Pediatrics, University of New Mexico Health Sciences Center, Albuquerque, NM 87131-0001 USA; 4 Department of Medicinal Chemistry, University of Minnesota College of Pharmacy, Minneapolis, MN 55455 USA

**Keywords:** Neuropathic pain, Prenatal alcohol exposure, Glia, Neuroimmune function, Peripheral immune system, Spinal cord

## Abstract

Recently, moderate prenatal alcohol exposure (PAE) was shown to be a risk factor for peripheral neuropathy following minor nerve injury. This effect coincides with elevated spinal cord astrocyte activation and ex vivo immune cell reactivity assessed by proinflammatory cytokine interleukin (IL) -1β protein expression. Additionally, the β2-integrin adhesion molecule, lymphocyte function-associated antigen-1 (LFA-1), a factor that influences the expression of the proinflammatory/anti-inflammatory cytokine network is upregulated. Here, we examine whether PAE increases the proinflammatory immune environment at specific anatomical sites critical in the pain pathway of chronic sciatic neuropathy; the damaged sciatic nerve (SCN), the dorsal root ganglia (DRG), and the spinal cord. Additionally, we examine whether inhibiting LFA-1 or IL-1β actions in the spinal cord (intrathecal; i.t., route) could alleviate chronic neuropathic pain and reduce spinal and DRG glial activation markers, proinflammatory cytokines, and elevate anti-inflammatory cytokines. Results show that blocking the actions of spinal LFA-1 using BIRT-377 abolishes allodynia in PAE rats with sciatic neuropathy (CCI) of a 10 or 28-day duration. This effect is observed (utilizing immunohistochemistry; IHC, with microscopy analysis and protein quantification) in parallel with reduced spinal glial activation, IL-1β and TNFα expression. DRG from PAE rats with neuropathy reveal significant increases in satellite glial activation and IL-1β, while IL-10 immunoreactivity is reduced by half in PAE rats under basal and neuropathic conditions. Further, blocking spinal IL-1β with i.t. IL-1RA transiently abolishes allodynia in PAE rats, suggesting that IL-1β is in part, necessary for the susceptibility of adult-onset peripheral neuropathy caused by PAE. Chemokine mRNA analyses from SCN, DRG and spinal cord reveal that increased CCL2 occurs following CCI injury regardless of PAE and BIRT-377 treatment. These data demonstrate that PAE creates dysregulated proinflammatory IL-1β and TNFα /IL-10 responses to minor injury in the sciatic-DRG-spinal pain pathway. PAE creates a risk for developing peripheral neuropathies, and LFA-1 may be a novel therapeutic target for controlling dysregulated neuroimmune actions as a consequence of PAE.

## Introduction

Fetal alcohol spectrum disorder (FASD), a condition that occurs as a result of prenatal alcohol exposure (PAE), results in cognitive and behavioral deficits [32]. Unfortunately, many of the central nervous system (CNS) deficits are not always apparent. By using animal models of FASD, many of the insidious effects of PAE have been uncovered. Interestingly, a growing body of evidence shows that PAE alters immune function and reactivity. Specifically, the viability of microglial cells within the cerebellum is decreased as a consequence of developmental ethanol exposure [22]. Additionally, a separate study demonstrates that hippocampal glial cells are increased in their activation state, which coincides with heightened expression of the proinflammatory cytokine interleukin (IL)-1β [57]. These and other PAE studies utilize high levels of alcohol exposure to mimic the effects of binge alcohol consumption, leaving the question of whether low-to-moderate levels of PAE adversely impact the function of the central nervous system in adulthood. Recent reports investigated whether spinal cord pain processing is exaggerated by the effects of PAE on glial and proinflammatory cytokine actions. These studies demonstrate that moderate PAE enhances both peripheral and spinal immune cell and proinflammatory cytokine action. That is, immune responses become ‘primed’ (i.e. sensitized) such that applying a sciatic nerve (SCN) injury generates potentiated microglial and astrocyte responses at the spinal cord, as well as enhanced expression of proinflammamtory cytokines, such as tumor necrosis factor-α (TNFα) and IL-1β, at the damaged SCN [41, 42]. Additionally, blunted expression of the anti-inflammatory cytokine IL-10, which suppresses neuropathic pain [24, 61], is observed in the dorsal root ganglia (DRG) and the SCN in PAE rats compared to non-PAE controls [42]. These results support the possibility that PAE results in susceptibility to pro-nociceptive proinflammatory neuroimmune responses well into adulthood.

In an effort to address the possible risk of developing neuropathy created by PAE, the injury to the SCN was substantially reduced in an effort to unmask the effects of moderate PAE on glial and immune priming [49]. This reduction uncovered an alteration in spinal astrocytes, as the astrocyte marker, glial fibrillary acidic protein (GFAP) is significantly elevated in PAE rats with neuropathy [42, 49], while evidence for microglial activation was not observed. In support of a role for peripheral immune cells in sensitized responses to minor injury/challenge, heightened expression of TNFα and IL-1β was observed in leukocytes from PAE rats [49]. Together, these data suggest that the PAE leads to hyper-reactive neuroimmune response which may render one vulnerable to developing neuropathic pain.

Interestingly, the chemokine CCL2 and the β_2_-integrin, lymphocyte function-associated antigen-1 (LFA-1) are elevated in PAE rats [42]. CCL2 activates its cognate receptor CCR2, which is co-expressed with the surface receptor, intercellular adhesion molecule-1 (ICAM-1) on endothelial cells. Similarly, CCL2/CCR2 actions occur on cells that also express surface LFA-1, leading to an LFA-1 conformational change allowing for direct LFA-1/ICAM interactions to occur [11]. The LFA-1/ICAM interaction is a process underlying transendothelial leukocyte migration into CNS sites with increased CCL2 levels. LFA-1 is expressed by peripheral leukocytes (e.g. macrophages and T-cells) and in the CNS, by microglia [13, 17]. Following peripheral nerve damage, peripheral leukocytes utilize ICAM-1/LFA-1 interactions for the process of extravasation into the spinal cord where damaged nerve terminals release CCL2 and communicate to spinal pain projection neurons. Thus, elevated CCL2 expression occurs in the spinal regions where excitatory neuronal communication of the pain relays occur, further aiding in the induction of allodynia. Interestingly, LFA-1 expression can potentially influence IL-10 expression [12, 25]. A separate study looking at the influence of LFA-1 on cytokine activity demonstrated that pretreating cultured macrophage cells (RAW267) stimulated with lipopolysaccharide (LPS) using an LFA-1 antagonist, BIRT-377, results in a dose-dependent increase in IL-10 protein release with a simultaneous decrease in IL-1β and TNFα protein release [26]. Therefore, the present study examines whether blocking spinal LFA-1 activation can generate an anti-inflammatory phenotype within the DRG and spinal cord resulting in reversal of chronic allodynia in neuropathic susceptible PAE rats.

The present report utilizes a rodent model of peripheral neuropathy referred to as chronic constriction injury (CCI), but modified with significantly reduced injury by applying a single suture (minor CCI) rather than the established 4-suture paradigm [4]. By applying a minor CCI to a characterized rat model of moderate PAE, allodynia is induced only in PAE rats, replicating prior findings [49]. Following CCI, BIRT-377 is injected intrathecally followed by behavioral assessment. At maximal efficacy of BIRT-377 on suppression of allodynia, tissue is collected from the SCN, DRG, and dorsal horn of the spinal cord to characterize the proinflammatory cytokine and chemokine profile using IHC, electrochemiluminescence (for protein quantification assays), and mRNA gene expression. Additionally, IL-1 receptor antagonist (IL-1RA) is administered via the intrathecal (i.t.) route to determine whether spinal IL-1β is necessary for allodynia following minor injury. The goals of these studies will determine the SCN, DRG, and spinal immune factors altered and whether controlling the spinal cord cytokine/chemokine environment can reverse PAE induced sensitivity to allodynia.

## Materials and methods

### Animals and study group strategy

All procedures were approved by the Institutional Animal Care and Use Committee (IACUC) of the University of New Mexico Health Sciences Center and closely adhered to guidelines from the International Association for the Study of Pain for the use of animals in research. Long-Evans rat breeders purchased from Harlan Industries (Indianapolis, IN) were maintained in a breeding colony on a 12:12-h reverse light/dark schedule (lights on from 2100 to 0900 h), and fed Harlan Teklad rat chow and water, available ad libitum. For all experiments, 5–8-month old male prenatal alcohol or saccharin exposed (described below) rat offspring derived from 56 litters were used. Offspring were transferred to a standard light/dark cycle (lights on from 0600 h to 1800 h) and allowed to acclimate to these conditions for at least 30 days before commencing experiments. Additionally, rats were kept in these conditions for the duration of the study.

#### Moderate prenatal alcohol exposure using the voluntary drinking paradigm

Pregnant female rat dams are given either ethanol or saccharin throughout pregnancy until birth according to the voluntary drinking paradigm previously described [50]. Briefly, 3- to 4-month-old female Long-Evans rats are acclimated for 4 h per day, from 1000 to 1400 h (during their active phase), to drinking water containing 0.066% (*w*/*v*) saccharin (Sac) that gradually increased in ethanol content from 0% (*v*/v) on Days 1–2, to 2.5% (v/v) on Days 3–4, to 5% (v/v) on Day 5 and thereafter for two weeks. It should be noted that regular drinking water is available at all times during housing. Thus, animals could voluntarily choose to drink either saccharin sweetened water containing 5% ethanol or regular water during the 4-h drinking period. At the end of the 2-week pre-pregnancy drinking phase, the mean daily ethanol consumption is determined for each rat and rats that consumed 1 standard deviation below the group mean are excluded from the study. Subsequently, female rats are assigned to either a 5% ethanol or Sac control drinking group such that the mean pre-pregnancy ethanol consumption was similar between groups. The females are then placed with proven male rat breeders until pregnant (~ 2–5 days). No alcohol is consumed during the breeding period. Beginning on gestational day 1, rat dams are given either 0% (Sac) or 5% (PAE) ethanol in Sac water (4 h/day, during the active phase). Sac rats are given a volume of 0% ethanol in Sac water that is matched to the mean volume voluntarily consumed by the 5% ethanol group. Total ethanol consumption is recorded for each dam, which averaged 2.04 g of ethanol/kg body weight/day. This level of drinking by rat dams produced a mean peak serum blood alcohol concentration of 60.8 mg/dl [50]. No significant differences are observed between prenatal treatment groups in dam weight gain during pregnancy, pup birth weights, or litter size, replicating previous reports [42, 49, 50]. Offspring are weaned at 24 days of age and male offspring pair-housed with the exception of five single-housed rats due to incompatibility with a cage mate. For all experiments, 5–8-month-old prenatal alcohol or saccharin-exposed animals are used. Behavioral testing of offspring is performed during the first three hours of the light cycle (inactive phase) to avoid the influence of elevated hormones under normal circadian rhythms.

A total of 108 male offspring are used in all experiments. Offspring are predominantly pair-housed with 5 rats single-housed due to incompatibility with a cage mate. Rat offspring are split into three groups. It should be noted that none of the experimental groups contained more than one subject from a given litter to avoid “litter effects”. The first group and second group consists of 43 (24 with prenatal saccharin exposure, Sac; and 19 with prenatal alcohol exposure; PAE) and 51 (23 Sac and 28 PAE) rats, respectively, and are used for behavioral examination of hindpaw sensitivity following i.t. treatment of BIRT-377 and its effects on neuropathy produced by minor peripheral nerve injury. Following behavioral verification for allodynia, Group 1 rats are used for protein and mRNA analysis whereas Group 2 rats are used for spinal cord and DRG immunohistochemical (IHC) analysis. Group 3 consisted of 14 (PAE) rats and are used to examine the effects of an i.t. administration of IL-1 receptor antagonist (IL-1RA) on CCI-induced allodynia. In all cases, experimenter is blinded to the group identities of all experimental conditions.

### Chronic constriction injury (CCI)

In adult male Sac or PAE rats, sham or CCI aseptic surgical procedures are performed as previously described [4, 42, 49] and modified, as described here. Under isoflurane anesthesia (3% volume in oxygen), the sciatic nerve is carefully isolated with sterile glass prongs and snuggly ligated with 1 (minor CCI) segment of sterile 4–0 chromic gut suture (Ethicon, Somerville, NJ) without pinching into the nerve. Sterile isotonic saline (0.9%) is applied to the nerve during the procedure to prevent dehydration. Sham surgery involves isolation of the sciatic nerve similar to CCI but without nerve ligation. The nerve is then gently placed back into position and the overlying muscle is sutured closed with two 3–0 sterile silk sutures (Ethicon, Somerville, NJ). Rats fully recover from anesthesia within approximately 5 min and are monitored daily following surgery for any post-operative complications. Two rats (1.8%) were excluded from the study due to the presence of hindpaw autotomy.

### Behavioral assessment of allodynia

Allodynia was assessed using the von Frey fiber test as previously described [10, 49]. Briefly, habituation to the testing environment required placing rats atop 2-mm thick parallel bars spaced 8-mm apart allowing full access to the plantar hindpaw. Habituation occurres for approximately 45 min/day for 4 sequential days within the first 3 h of the light cycle (inactive phase) in a sound- and temperature-controlled dimly lit section of the home colony room. Baseline (BL) responses are then assessed using the von Frey behavioral test. A total of 13 calibrated monofilaments are used in these studies. Additionally, these calibrated monofilaments (3.61–5.18 log stimulus intensity) are applied to the plantar surface of the left and right hindpaw for a maximum of 8 s per application. Random order between left and right hindpaw assessment is conducted. A metronome placed in the room provided guidance at 1 tick/sec. Lifting, licking, or shaking of the paw is considered a response. In a similar manner to BL evaluation, one group of rats is re-assessed following CCI or sham surgery through Day 10 prior to drug treatment. A second group of rats is assessed through Day 28 prior to drug treatment. The experimental tester was blind to the prenatal and surgical treatment groups. At either Day 10 or Day 28, rats are given an i.t. injection of drug or vehicle (described below) and behavioral re-assessment occurs daily for 4 sequential days post-injection to examine the effects of i.t. BIRT-377, or at 1, 2, 2.5, 3, and 24 h post-injection to examine the effects of i.t. IL-1RA.

### Drug preparation

BIRT-377 is a small molecule characterized as a non-competitive inhibitor of leukocyte function-associated antigen-1 (LFA-1) that minimizes the active conformation of this transmembrane β_2_-adhesion receptor expressed on leukocytes (e.g. T cell and myeloid cells). BIRT-377 (Tocris, Bristol, UK) was initially reconstituted in 100% ethanol as a stock solution at a concentration of 22.156 mg/ml, aliquoted into 0.5 ml sterile Eppendorf tubes and stored in a clean sealed container at 4 °C for later use. On the day of i.t. injection, sterile ddH_2_0 is warmed in a water bath and BIRT-377 is added to reach a concentration of 500 ng/20 μl for i.t. injection and vortexed for 2 min. Vehicle consisted of sterile ddH_2_O. Animals are injected within the hour following drug dilution.

IL-1 receptor antagonist (IL-1RA) (R&D systems, Minneapolis, MN) is reconstituted in sterile ddH_2_0 at a stock concentration of 50 mg/ml, aliquoted into 0.5 ml sterile Eppendorf tubes and stored at − 20 °C for later use. On day of i.t. drug injection, IL-1RA is diluted in 0.1% rat serum albumin (RSA) in sterile phosphate buffered saline (PBS) to a concentration of 1 μg/μl containing 0.01% RSA. Therefore, each rat received 20 μg of IL-1RA in 20 μl i.t. Vehicle consists of 0.1% RSA in sterile PBS.

### Intrathecal (i.T.) injection

BIRT-377, IL-1RA or vehicle is administered using an acute i.t. catheter on Day 28 (BIRT-377) or Day 10 (BIRT-377 or IL-1RA) post-surgery as described previously [36]. Briefly, under isoflurane anesthesia (3% volume in oxygen), the beveled needle end of an 18-gauge sterile (Bectin Dickson, Franklin Lakes, NJ) needle with the plastic hub removed is inserted between lumbar vertebrae L5-L6. Because the beveled tip of the needle is wider than L5-L6 intervertebral foramen, only ~ 1 mm of the beveled needle tip is capable of puncturing the dura mater to access the underlying arachnoid space. Consequently, spinal tissue or rootlets from the cauda equina remain uninjured and intact. PE-10 tubing (Fisher Scientific, Hampton, NH) is fitted with a 30-gauge sterile needle (Bectin Dickson, Franklin Lakes, NJ) containing a Hamilton syringe at one end and labeled with a marking at 7.7 cm at the opposite end. Drug or equivolume of vehicle (20 μl) is withdrawn from the open side of the PE-10 tubing. The open end of the tubing is then threaded into the 18-gauge needle until the 7.7 cm marking is reached. This position corresponds to the lower lumbar L4-L5 region of the spinal cord. Correct placement of the PE-10 is determined by twitching of the hindleg and/or tail during catheter advancement, as well as cerebrospinal fluid efflux from the externalized portion of the needle. Drug is administered over a 25 s interval. Immediately following drug or vehicle administration, tubing is removed followed by removal of the guide 18-g needle and rats are allowed to recover before being placed in their home cage. Rats fully recover from anesthesia within 5 min and 100% motor recovery is observed following injection.

### Immunohistochemical (IHC) tissue sample preparation

All tissues from rats behaviorally verified for allodynia on Day 4 (Either Day 14 or Day 32 post-surgery) after i.t. BIRT-377 injection are collected for IHC processing as described previously [42, 49]. Briefly, rats are overdosed with sodium phenobarbital (390 mg/ml Sleepaway, Fort Dodge Animal Health, Fort Dodge, IA) and transcardially perfused with 0.1 M phosphate buffered saline (PBS; pH = 7.4), initially at a rate of 28 ml/min, then increased to 32 ml/min (~ 10 min), followed by 4% paraformaldehyde (PFA; pH = 7.4; 28–34 ml/min, 8 min). Immediately following transcardial perfusion, the spinal vertebral column from C2-L6 with the spinal cord intact within the vertebral column are collected and cut at vertebra T7 into a rostral and caudal half of the spinal vertebral column. All spinal cord sections undergo 48-h post-fixation in 4% PFA at 4 °C.

Following post-fixation, the entire spinal cord (T7- L6) is carefully isolated, and separately, the corresponding L4-L6 dorsal root ganglia (DRG), making sure to maintain tissue integrity. The isolated spinal cords are then subsequently paraffin processed. At a later time, DRGs are individually dissected and paraffin processed. Paraffin-embedded blocks are then subsequently sliced on a microtome with adjacent 7 μm tissue sections mounted onto vectabond-treated slides.

To investigate spinal augmented glial cell activation and cytokine profile, the expression of the astrocyte marker, glial fibrillary acidic protein (GFAP), the microglial activation marker ionized calcium-binding adapter molecule 1 (Iba1), the proinflammatory cytokine marker IL-1β, and the anti-inflammatory marker IL-10 in L4–6 spinal segments, as reported previously [61]. Slides containing tissue from lumbar (L4-L6) spinal cord and (L4-L6) DRG are chosen for staining to capture overall reactivity [61]. Paraffin-processed tissues undergo deparaffinization followed by rehydration and antigen retrieval procedures in a standard rice cooker at 94–96 °C allowing for gentle and equal distribution of temperature warming throughout tissue sections. For antigen retrieval, polyclonal GFAP required a Tris-based buffer at pH 9.5 (BioCare Medical, Concord, CA), Monoclonal GFAP, Iba1, and IL-1β required a Tris-based buffer at pH 9.0 (Vector, Burlingame, CA), and IL-10 required a citrate-based buffer at pH 6.0 (BioCare Medical, Concord, CA). All tissue sections are incubated with 5% normal donkey serum (NDS), PBS pH 7.4 for 2 h, followed by overnight primary antibody incubation using rabbit anti-rat polyclonal GFAP (Millipore, 1:1000), rabbit anti-rat monoclonal GFAP (Abcam. 1:400), rabbit anti-rat polyclonal Iba-1 (Wako, 1:300), or rabbit anti-rat polyclonal IL-1β (Santa Cruz, 1:100), or goat anti-rat polyclonal IL-10 (Vector Labs, 1:250) in a humidity chamber at 4 °C. Tissues are washed 3x with 0.1 M PBS pH 7.4. All tissues, except those containing IL-10 antibody, are incubated with donkey-anti rabbit FITC- or TRITC- conjugated secondary antibody incubation for 2 h in a humidity chamber at room temperature and rinsed in 0.1 M PBS. IL-10 spinal cord and DRG sections are incubated with a biotinylated donkey anti-goat secondary antibody (Vector Labs, 1:1300) for 1 h followed by a 1 h incubation with Vectastain ABC Elite kit (Vector Labs, Burlingame, CA). Sections are incubated for 10 min with TSA plus Fluorescein System (PerkinElmer Life Sciences, Waltham, MA,). All tissues are stained with the nuclear stain 4,6-diamidino-2-phenylindole (DAPI) (Vector Labs, Burlingame, CA) separately before cover slipping. These slides are left at room temperature overnight and then placed at 4 °C until proceeding with image acquisition and microscopy analysis [49, 61].

### Microscope spectral imaging for immunofluorescent quantification

Image acquisition for spectral analyses is performed using the Nuance spectral imaging system (Perkin Elmer, Waltham, MA) as described previously [42, 49]. Briefly, images of dorsal horn spinal cord are obtained using either a 20X or 40X (IL-10 spinal cord sections) objective with a Zeiss Axioplan2 inverted fluorescence microscope. A flat-field correction is applied to correct for uneven illumination during image acquisition. Image cubes are obtained from multi-labeled tissue containing DAPI, conjugated secondary antibody, as well as autofluorescence. A spectral library is then created using single-labeled slides for each fluorophore (e.g. FITC or TRITC) and a label-free (autofluorescence) slide. A computed spectrum (420–720 nm) is obtained by separating the known spectrum (autofluorescence) from mixed spectrum (single labeled) to produce a pure spectrum of each fluorophore. This allowed the separation of spectrum from multi-labeled slides (e.g. DAPI + TRITC) to obtain composite images containing only labels of interest. These composite images are then used for further analysis using Slidebook 6 software (below). For dorsal horn spinal cord sections ipsilateral to sciatic manipulation, sixteen to thirty-two images per experimental group (4 sections per animal, 4–8 animals per condition) were acquired and analyzed. For L4-L6 DRG sections ipsilateral to the sciatic manipulation, thirty images per experimental group (6 sections/rat, 5 rats per condition) were acquired and analyzed. It should be noted that the 6 sections per animal were made up of the L4, L5, and L6 DRG and contained two images per DRG level. Representative acquired images from the Nuance spectral imaging system from the dorsal horn spinal cord of GFAP immunoreactive and Iba1 immunoreactive regions are shown in Fig. 2c-d, as well as IL-1β and IL-10 shown in Fig. 3c-d; and in DRG of satellite glial cells (SGCs), IL-1β, and IL-10 in Fig. 5. 20x and 40x objectives are used to produce representative images.

### SlideBook6 software image analysis

Composite images are analyzed using SlideBook6 software (Intelligent Imaging Innovations, Denver, CO, USA) as described previously [49]. Briefly, to discriminate signals originating from artifacts, the experimenter determined an acceptable threshold of very low-level emission fluorescence intensity per each experimental condition (polyclonal GFAP and Iba1 analysis), or per individual animal (monoclonal GFAP, IL-1β and IL-10 analysis). This was done by closely replicating the on-screen composite computer image with that observed through the microscope eyepiece, as described previously [42, 49]. In addition, for spinal cord sections, the dorsal horn (region of interest (ROI)) is outlined for analysis eliminating the surrounding white matter and peri-spinal blank space (intrathecal space). For DRG sections, ROI included all cell bodies found within the composite image. The image is refined to include the predetermined threshold within the outlined area. The ‘Sum Intensity’ (the total signal within the outlined area in the dorsal horn) is determined and divided by the ‘Area’ (total area in micrometers squared (μm^2^)) to obtain the ‘Fluorescence Intensity’. The average of the adjacent sections from a single slide (representing a single animal) is calculated to determine the value for each animal. Finally, averages for each slide within the same condition are calculated along with the standard deviation and standard error of the mean. Data are analyzed as “Sum Intensity of ROI/μm^2^”.

### Tissue collection and total RNA isolation

Following behavioral assessment on Day 4 post-injury (Day 14 post-surgery), rats are deeply anesthetized with isoflurane (induction 3% vol. followed by 5% in oxygen) for 10–12 min. Rats are then transcardially perfused with ice cold 0.1 M PBS pH 7.4 for 3 min at a rate of 24 ml per minute. The perfused body is placed on a frozen gel refrigerant pack (Glacier Ice, Pelton Shepherd Industries, Columbus, OH). Laminectomy and tissue dissection is performed to collect both ipsilateral and contralateral lumber L4-L6 spinal cord, followed by L4-L6 dorsal root ganglia, and finally ipsilateral sciatic nerve. All samples are placed in DNase/RNAse-free 1.5 ml centrifuge tubes (VWR International, Radnor, PA), flash frozen on dry ice, and stored at − 80 °C for future analysis.

Flash frozen tissues are transferred to ice and 100 μl sterile 1 x PBS was added. It should be noted that, in contrast to spinal cord and DRG tissue, sciatic nerve is quickly chopped with scissors for ~ 2 min on ice. All tissues are then homogenized using a motorized VWR disposable Pellet Mixer and motor pestle system (VWR International, Radnor, PA) for about 30 s. 60% of the tissue homogenate is then processed for protein analysis (described below). The remaining 40% of the homogenate is added to chilled Qiazol Lysis Reagent (Qiagen, Hilden, Germany) for total RNA isolation (described below). It should be noted that, due to the small quantity of DRG tissue collected, 100% of tissue homogenate is used for RNA isolation and not protein analysis.

### Tissue lysate preparation and Meso scale discovery (MSD) multi-spot assay system

Flash frozen ipsilateral sciatic nerve and ipsilateral and contralateral lower lumbar L4-L6 spinal cord sections collected from animals are prepared for MSD Multi-Spot Assay system as previously described [42]. Briefly, tissues are allowed to thaw on ice and homogenized using a Fisherbrand Disposable Pestle system (Fisher Scientific, Hampton, NH). Samples are then sonicated in buffer containing protease inhibitors per instructions of manufacturer. All samples are then centrifuged at 4200 g, at 4 °C, for 10 min. Lysate is collected from samples and Bradford protein assay (Biorad, Hercules, Ca) is used to determine protein concentrations. Using a V-PLEX immunoassay (MesoScale Discovery, Gauthersburg, MD), 100 μg protein of all samples is used to determine cytokine expression levels for TNFα, IL-1β, IL-10, and CXCL1 as previously validated [33, 42, 47].

### Total RNA isolation and mRNA analysis by quantitative real-time PCR

Total RNA is extracted from ipsilateral sciatic nerve, ipsilateral and contralateral spinal cord, and ipsilateral DRG. Tissue is placed in chilled Qiazol Lysis Reagent (Qiagen, Hilden, Germany) and homogenized for 15 s. Total RNA is then extracted using a miRNeasy Micro Kit (Qiagen, Hilden, Germany) per manufacturer’s instructions. It should be noted that an additional buffer RPE and 80% EtOH wash steps is added to remove any additional salt contaminants added from the PBS step. Tissue is centrifuged at 15,000 rpm for 1.5 min at 4 °C. PBS is aspirated and 35 μl of protease inhibitor solution (MesoScale Discovery System) is added and samples were stored on dry ice and later stored at − 80 °C for future analysis (described below).

RNA concentration and quality is assessed using Nanodrop (ThermoFisher Scientific, Walham, MA). RNA samples are then diluted to standardized RNA concentrations of 195 ng/μl for ipsilateral DRG, 137 ng/μl for ipsilateral and contralateral lumbar spinal cord, and 100 ng/μl for ipsilateral sciatic nerve. Standardized samples are then transcribed to cDNA at 2145 ng for ipsilateral DRG, 950 ng for ipsilateral SCN, and 1438.5 ng for ipsilateral and contralateral lumbar spinal cord. Reverse transcription is performed for all tissue types using a Superscript IV VILO cDNA Synthesis Kit (Invitrogen, Carlsbad, CA).

Levels of mRNA expression is measured and analyzed as described previously [35]. For assessment of transcripts, a dilution factor of 1:2.5 is applied to cDNA samples for ipsilateral DRG, 1:2 for ipsilateral SCN, and 1:2.2 for ipsilateral and contralateral lumbar spinal cord. cDNA dilution for assessment of rat 18S rRNA normalizer is 1:200 for all tissue types. Levels of mRNA and “Normalizer” rat 18 s rRNA (Rn18s, Taqman Assay ID#: Mm03928990_g1), is assayed in triplicate using quantitative real-time PCR (qRT-PCR) with Taqman Gene Expression Assays (ThermoFisher Scientific, Waltham, MA). Gene expression assays are deemed to be “best coverage” assay by the manufacturer and is analyzed with the formula C = 2^CT^Normalizer^/2^CT^Target^ in order to exclude detection of genomic DNA [31, 51]. Chemokines chosen for qRT-PCR include CCL2 (Taqman Assay ID#: Rn00580555_m1), CXCL10 (Taqman Assay ID#: Rn01413889_g1), CXCL1 (Taqman Assay ID#: Rn00578225_m1), and CX3CL1 (Taqman Assay ID#: Rn00593186_m1). These chemokines have been previously shown to be upregulated during neuropathic pain conditions [2, 21, 29, 64], and additionally, CCL2 and CX3CL1 have been shown to be altered following PAE [42, 46].

### Statistical analysis

SPSS (IBM, Chicago, IL, USA) is used for all behavioral analysis. At baseline (BL), a 3-way (2 × 2 × 2) analysis of variance (ANOVA) is applied to assess differences between prenatal exposure (Sac versus PAE), surgical treatment (Sham versus minor CCI) and drug injection (vehicle versus BIRT-377). Additionally, a 3-way (2 × 3) repeated measures ANOVA is used for analysis of the between-subject factors of prenatal exposure, surgery, and injection for days post-surgery as well as days post-injection. Furthermore, 3-way (2 × 2 × 2) ANOVA is used for analysis of all IHC immunoreactivity data. Data acquired from V-Plex immunoassay and qRT-PCR are analyzed using GraphPad Prism version 7 software (GraphPad Software Inc., San Diego, CA, USA). *Post-hoc* examination of IHC immunoreactivity is analyzed using Fisher’s LSD test. The threshold for statistical significance was set a priori at α = 0.05 for all sets of multiple comparisons. In order to minimize unnecessary duplication, we use the minimum number of animals possible to make statistically significant conclusions, which is based on our previous publications [10, 42, 49, 60, 61]. Outliers were removed following Grubbs’ Z-test [16]. In all cases, the data are presented as the mean ± SEM. All figures are created in GraphPad Prism version 7 software (GraphPad Software Inc., San Diego, CA, USA).

## Results

### I.T. Injection of BIRT-377 reverses enduring allodynia resulting from minor CCI in PAE rats

**Fig. 1:**
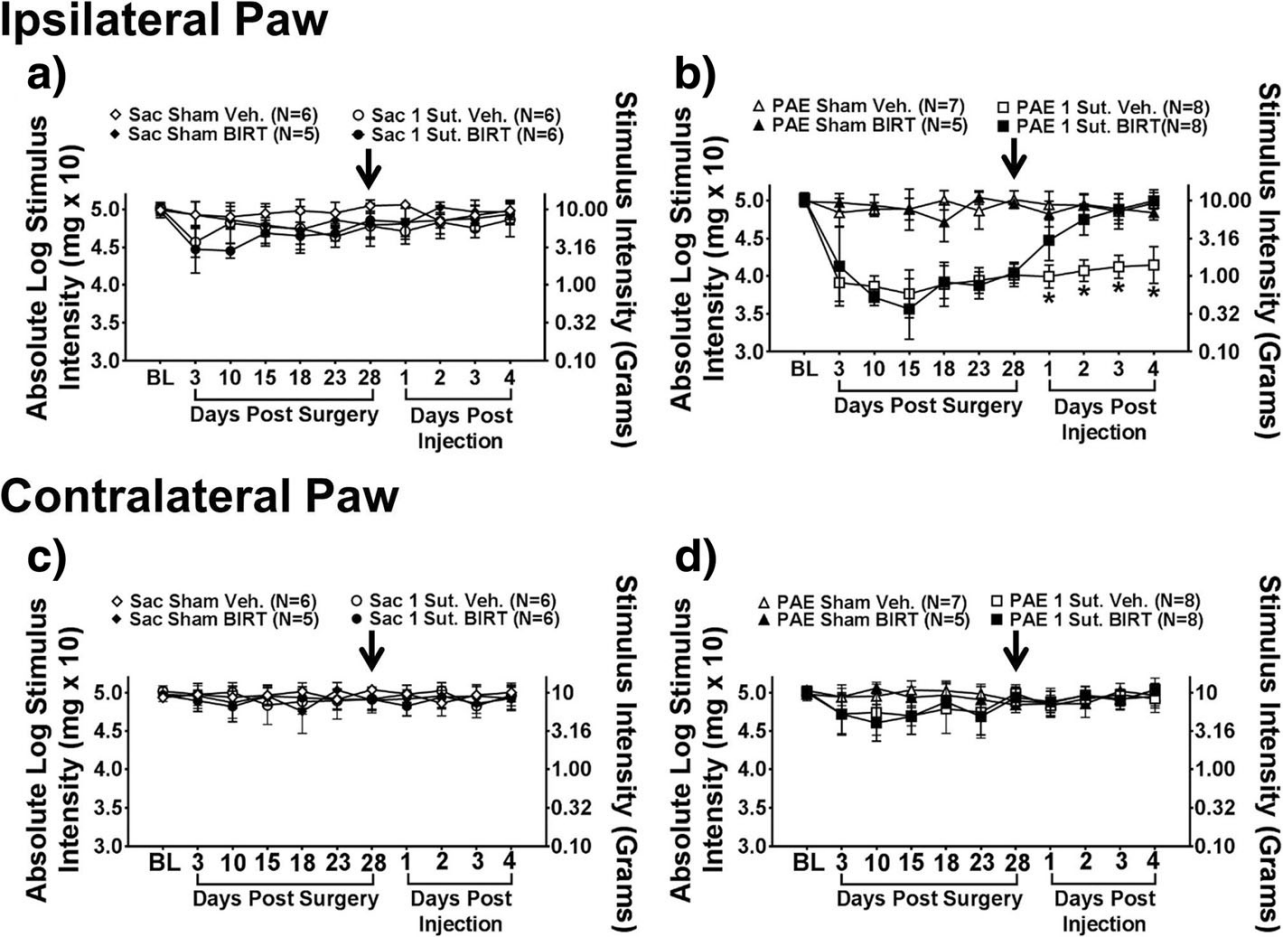
Enduring allodynia from minor injury in PAE rats is controlled by i.t. BIRT-377. Ipsilateral and contralateral hindpaw response thresholds from minor sciatic nerve injury (CCI) in adult PAE and Sac control rats. **a-b** At baseline (BL), rats displayed no differences in hindpaw sensitivity. **a-b** Following minor CCI, Sac control rats do not develop increased sensitivity whereas PAE rats develop enduring unilateral allodynia up to Day 28 post-surgery. **c-d** No changes in contralateral hindpaw sensitivity is observed following sham or CCI in Sac or PAE rats. **a-b** At Day 28, all animals received either vehicle or BIRT-377. **a-b** Following injection of BIRT-377, Sac rats maintain normal sensitivity. In contrast, allodynic rats with PAE return to normal sensitivity compared to vehicle injected rats. Sensitivity in the contralateral paw remained normal in all rat groups. *N* = 5–8 rats per group. Arrow indicates time of i.t. injection

Previous studies show that PAE-induced sensitivity to allodynia is enduring for at least 28 days after CCI [42]. Thus, we want to determine whether blocking the activated LFA-1 altered enduring PAE induced sensitivity to allodynia. To accomplish this, i.t. BIRT-377 is administered at Day 28 post-surgery. Interestingly, all rats reveal similar hindpaw sensitivity at BL (ipsilateral, F_1,43_ = 1.568, *p* = 0.217), indicating that PAE alone does not induce allodynia, but rather, it is not until a second insult is applied that the effects of PAE are unmasked, which replicates prior findings [42, 49, 63] (Fig. 1). Following CCI, a main effect of alcohol exposure (ipsilateral, F_1,43_ = 152.120, *p* < 0.0001), surgery (ipsilateral, F_1,43_ = 404.296, *p* < 0.0001) and an interaction between alcohol exposure and surgery (ipsilateral, F_1,43_ = 170.740, *p* < 0.0001) is observed. Normal sensitivity is maintained throughout the 28-day time-course following sham or minor (1-suture) CCI surgery in healthy Sac control rats (Fig. 1a). In contrast, compared to PAE rats with sham surgery that maintain normal sensitivity through day 28 post-surgery, minor CCI results in robust and enduring unilateral allodynia (Fig. 1b) (ipsilateral, F_1,43_ = 165.47, *p* < 0.0001). At Day 28, all rats received BIRT-377 or vehicle injection where a significant interaction is seen in the ipsilateral hindpaw between alcohol exposure, surgery, and injection (ipsilateral, F_1,43_ = 26.280, *p* < 0.0001). Chronic allodynia is reversed in PAE rats with hindpaw responses similar to BL levels following BIRT-377, with maximal reversal seen by day 4 post-injection (Fig. 1b). No changes in hindpaw sensitivity are observed in sham groups or Sac-minor CCI. Additionally, no significant differences are seen in contralateral hindpaw responses (Fig. 1c-d) after surgery (F_1,43_ = 0.953, *p* = 0.334) and following injection (F_1,343_ = 0.181, *p* = 0.672). These data suggest that the blockade of activated LFA-1 may alter the pronociceptive glial and immune cytokine/chemokine spinal milieu such that a full reversal from chronic allodynia in PAE rats occurs.

### Spinal BIRT-377 treatment blunts spinal glial activation

**Fig. 2:**
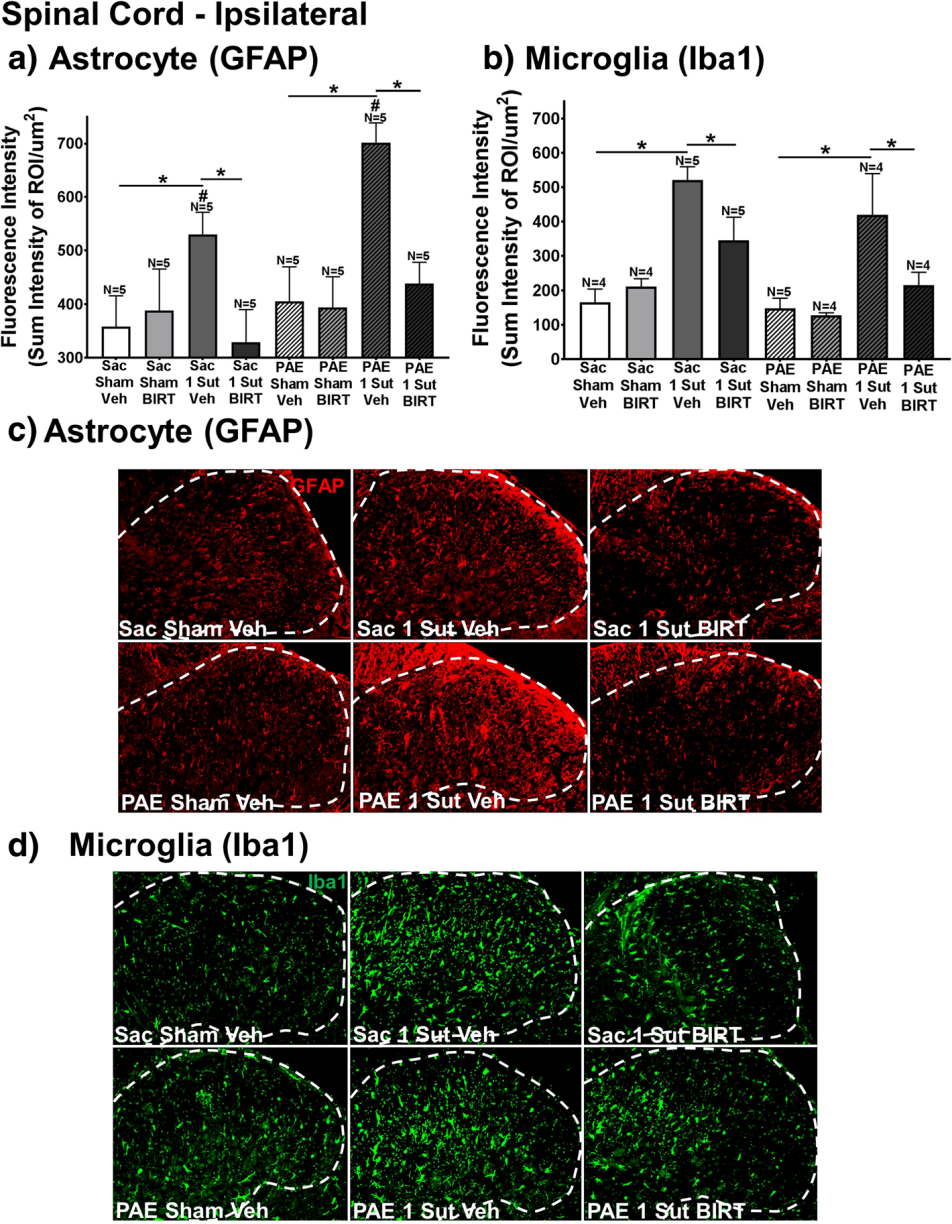
Glial activation is significantly decreased following i.t. BIRT-377 injection in rats with enduring allodynia. **a-d** At Day 4 post-injection (Day 32 after minor CCI) spinal cord tissues is harvested for quantification of immunohistochemical (IHC) immunoreactivity for (**a**) astrocyte activation (GFAP) and (**b**) microglial activation (Iba1) within the ipsilateral dorsal horn of the spinal cord (rats from data shown in Fig. 1). Fluorescence intensity is defined as the total immunoreactivity within the dorsal spinal cord [identified as a region of interest (ROI)] divided by the area of the ROI. **a-b** Following minor CCI, both PAE and Sac rats display significant increases in GFAP and Iba1 immunoreactivity compared to their corresponding sham groups, with spinal cords from PAE/1-sut rats revealing the most robust GFAP increases. BIRT-377 resulted in significant decreases in both GFAP and Iba1 immunoreactivity in rats with minor CCI. Minor CCI PAE rats reveal elevated GFAP immunoreactivity compared to Sac rats with minor CCI. Representative images of (**c**) GFAP and (**d**) Iba1 immunoreactivity used in IHC analysis are shown for Sac/Sham/Veh, Sac/1-Sut/Veh, Sac/1-Sut/BIRT, PAE/ Sham/Veh, PAE/1-Sut/Veh, and PAE/1-Sut/BIRT at 20x. The white dashed line represents a portion of the superficial dorsal horn as is the ROI under examination. *N* = 4–8 rats per group. “1-sut CCI” = “minor CCI”. N = 4–5 rats per group. Asterisks indicate *p* < 0.05. Pound sign indicates significance amongst groups at *p* < 0.05

**Fig. 3:**
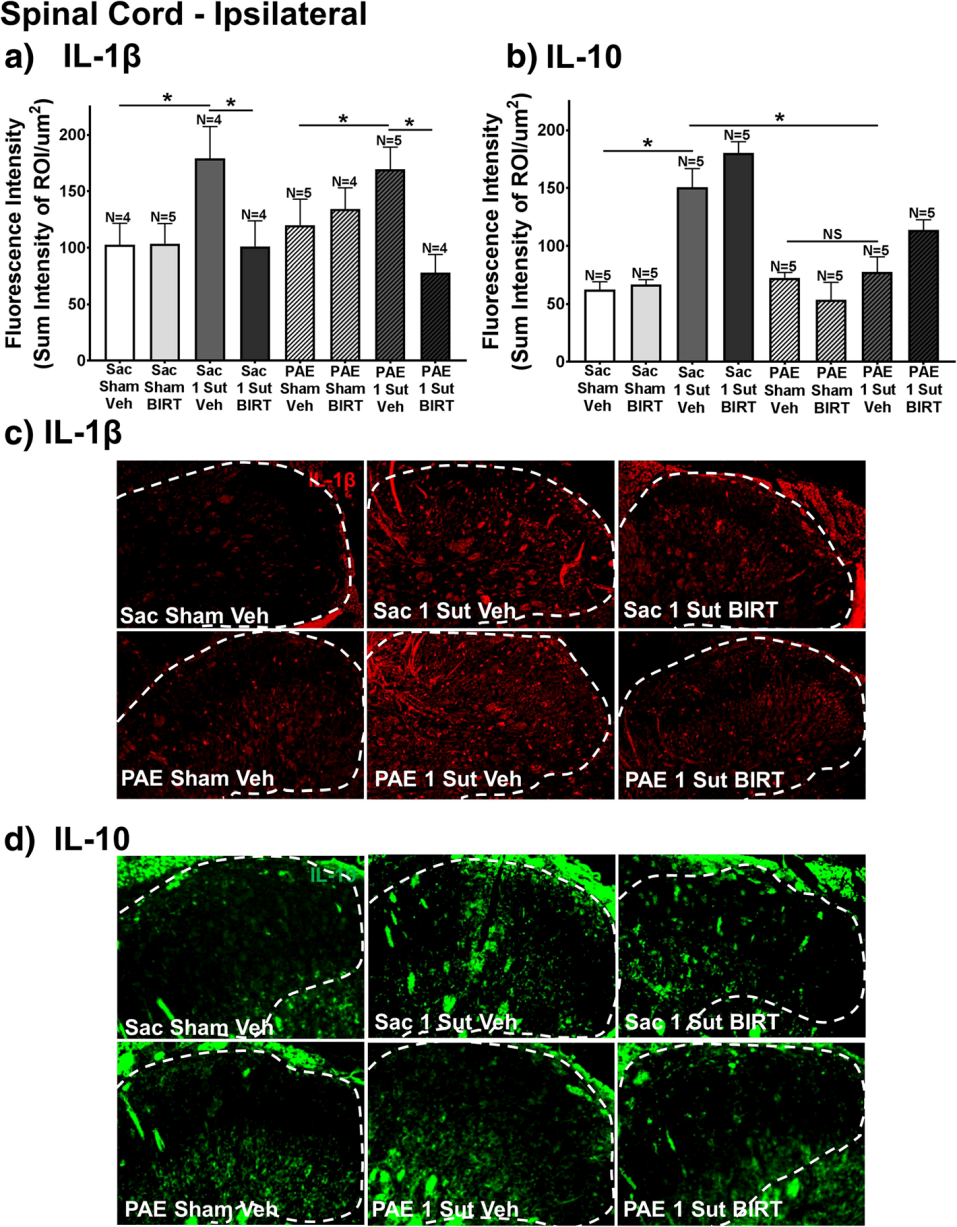
I.t. BIRT-377 injection significantly decreases IL-1β expression. **a-b** Separate analysis of IL-1β and IL-10 immunoreactivity within the dorsal horn spinal cord at Day 4 post-injection (Day 32 after minor CCI; rats from data shown in Fig. 1) reveal that IL-1β is significantly decreased compared to vehicle injected rats with minor CCI regardless of prenatal exposure. **b** Significant differences in IL-10 expression are observed in between Sac-sham rats and Sac-minor CCI rats given vehicle, whereas no differences are observed between all experimental conditions in PAE rats. Sac rats with minor CCI given vehicle reveal elevated IL-10 immunoreactivity compared to allodynic PAE rats given vehicle. PAE rats lack the compensatory IL-10 elevation to minor injury. Representative images of (**c**) IL-1β and (**d**) IL-10 immunoreactivity used in IHC analysis are shown for Sac/Sham/Veh, Sac/1-Sut/Veh, Sac/1-Sut/BIRT, PAE/Sham/Veh, PAE/1-Sut/Veh, and PAE/1-Sut/BIRT at either 20x for IL-1β and 40x for IL-10. The white dashed line represents a portion of the superficial dorsal horn as is the ROI under examination. “1-sut CCI” = “minor CCI”. N = 4–5 rats per group. Asterisks indicate *p* < 0.05

**Fig. 4:**
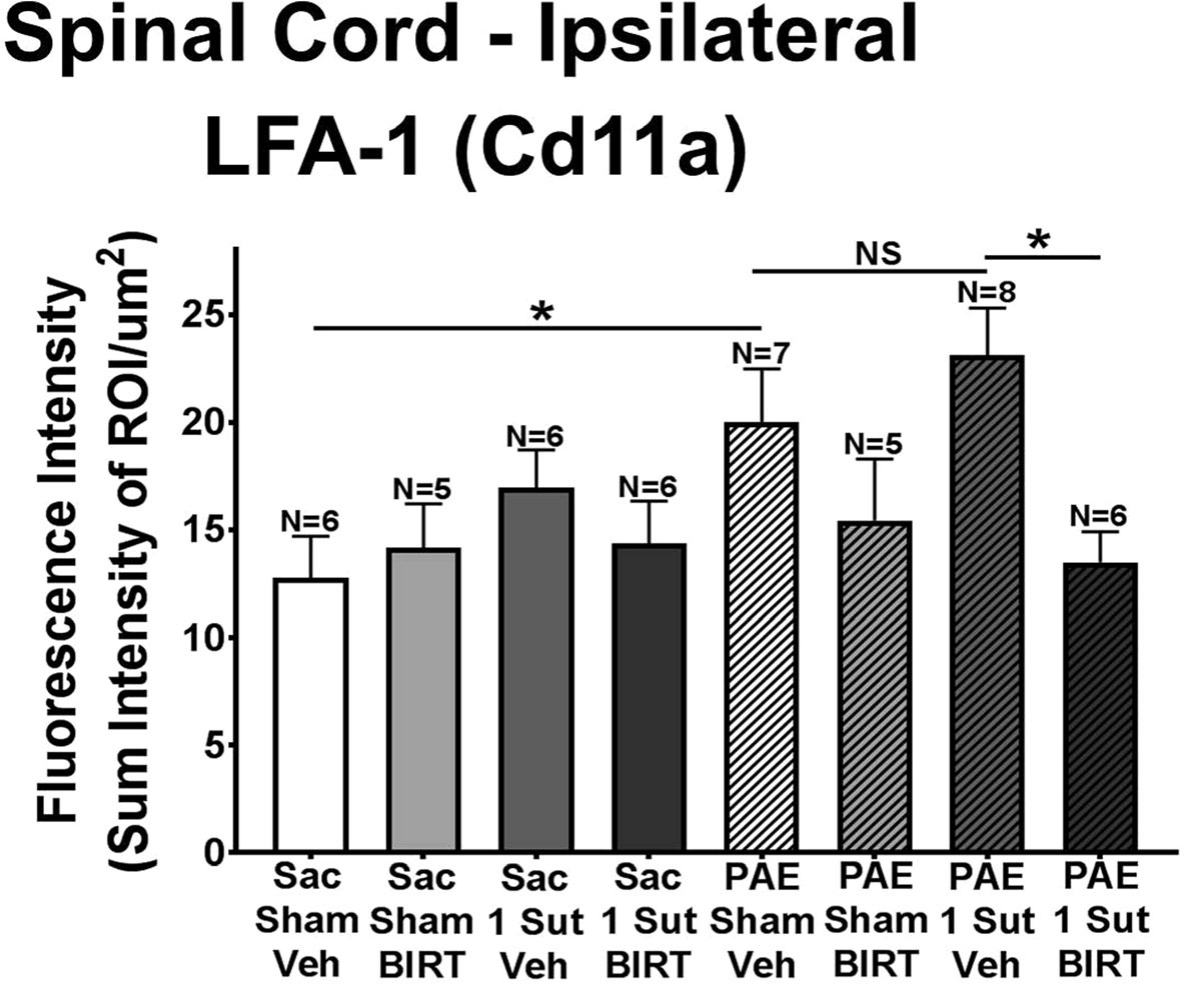
Increased basal levels of LFA-1 expression is present in PAE rats. Quantification of LFA-1 (Cd11a) immunoreactivity expression from behaviorally verified rats (Day 32 after minor CCI) (Fig. 1) reveal elevated expression of Cd11a observed in PAE- sham rats given vehicle compared to Sac-sham given vehicle. BIRT-377 injection resulted in significant decreases of Cd11a in PAE minor CCI rats. Additionally, no significant differences are observed between PAE rats given vehicle with sham or minor CCI. “1-sut CCI” = “minor CCI”. N = 5–8 rats per group. Asterisks indicate *p* < 0.05

To determine the mechanism by which BIRT-377 leads to allodynic reversal (Day 32 post-CCI surgery; Day 4 post-injection) in PAE rats, spinal cords are collected and processed for IHC and subsequent image analysis from all rat groups (rats from data shown in Fig. 1) for evaluation of glial activation (Fig. 2), cytokine expression (Fig. 3) and LFA-1 expression (Fig. 4) immediately following behavioral characterization of the effects of i.t. BIRT-377 (BIRT) on allodynia. Astrocyte and microglial activation demonstrate a significant interaction between surgery and injection (GFAP, F_1,32_ = 9.237, *p* = .005; Iba1, F_1,27_ = 6.775, *p* = 0.015). Analysis of GFAP immunoreactivity reveal elevated spinal astrocyte responses in Sac and PAE rats with minor CCI given a vehicle (Veh) injection compared to their corresponding sham groups (Fig. 2a), with the greatest increase of astrocyte activation observed from PAE rats with neuropathy induced by minor CCI. However, spinal cord astrocyte responses are decreased in spinal BIRT-377 treated rats (Fig. 2a), with the greatest magnitude decrease observed in PAE that revealed full reversal from allodynia. Interestingly, spinal microglial responses were increased in both PAE and Sac rats with minor CCI given vehicle compared to their corresponding sham groups (Fig. 2b) despite ongoing allodynia only in PAE rats. Spinal BIRT-377 injection results in significant decreases in microglial Iba-1 immunoreactivity (Fig. 2b). Given the lack of allodynia observed in Sac rats with minor CCI and the robust allodynia in PAE rats with minor CCI, the elevation in Iba-1 immunoreactivity may not reflect how microglia are playing a key role in the enduring allodynia observed only in PAE rats with minor CCI. Representative images (Fig. 2c-d) for GFAP and Iba1 are shown. These data suggest that PAE-induced allodynia requires mechanisms in addition to simply microglial activation. From these data, it is plausible that astrocyte actions play a greater role in PAE susceptibility to allodynia from minor injury.

### PAE blunts normal IL-10 responses following minor CCI

Both IL-1β and IL-10 immunoreactivity in the dorsal horn of the spinal cord are assessed (Fig. 3a-b) for changes following i.t. BIRT-377 injection (Day 32; rats from data shown in Fig. 1). Analysis of IL-1β and IL-10 immunoreactivity reveal an interaction between surgery and injection (IL-1β, F_1,27_ = 9.559, *p* < 0.001; IL-10, F_1,32_ = 7.066, *p* = 0.012). Compared to sham groups, PAE rats with minor CCI given vehicle reveal elevated levels of IL-1β (Fig. 3a). Interestingly, this elevation is also seen in Sac rats with minor CCI given vehicle injection despite the absence of allodynia (Figs. 1a and 3a). Furthermore, BIRT-377 is sufficient to significantly decrease spinal IL-1β immunoreactivity (Fig. 3a). In healthy Sac control rats, elevated levels of IL-10 immunoreactivity are measured following minor CCI given vehicle compared to Sac-sham rats given vehicle (Fig. 3b). Interestingly, PAE CCI rats with ongoing allodynia (e.g. given vehicle) do not show a significant increase in IL-10 immunoreactivity expression, an observation that is present in Sac CCI rats without allodynia. The elevated IL-10 immunoreactivity in Sac rats with CCI suggests the increase occurs in response to minor damage, possibly to maintain homeostasis and consequent non-neuropathy. In contrast, it is possible that in PAE rats, IL-10 fails to upregulate, thereby allowing for the development of allodynia following minor CCI (Fig. 3b). Intrathecal BIRT-377 results in slight non-significant increases in IL-10 immunoreactivity. Representative IHC images of IL-1β (Fig. 3c) and IL-10 (Fig. 3d) are shown. These results demonstrate that compared to Sac control rats following minor sciatic nerve injury, PAE alters healthy IL-10 responses important for controlling allodynia long after the minor insult has occurred (32 days after minor injury). Additionally, the data suggest that enduring allodynia in PAE rats following minor CCI (Fig. 1) may be a result of chronic increases in glial activation (Fig. 2) resulting in IL-1β expression (Fig. 3a) that goes unchecked by diminished IL-10 responses (Fig. 3b). Furthermore, the data demonstrate that BIRT-377 may reverse chronic allodynia by reducing glial activation (Fig. 2a-b) and decreasing IL-1β expression (Fig. 3a).

### LFA-1 expression is dysregulated in PAE rats

Immunoreactivity for Cd11a (LFA-1) is analyzed in the dorsal horn of the spinal cord in rats behaviorally verified for allodynia (Day 32; rats from data shown in Fig. 1). Data show a significant increase in LFA-1 immunoreactivity in PAE-sham rats given vehicle compared to Sac-sham rats given vehicle (Fig. 4) (*p* = 0.0188). Importantly, PAE-sham rats have not undergone a second insult, suggesting that the effects of PAE alone alter the LFA-1basal levels. No significant differences are observed between PAE-sham rats given vehicle and PAE-minor CCI given vehicle (Fig. 4). Additionally, a main effect of BIRT-377 (F_1,42_ = 5.529, *p* = 0.027) is revealed. It is possible that PAE the elevated basal expression of LFA-1 is a consequence of chronic aberrant ICAM-1 expression on vascular endothelial cells, which may be an underlying neuroimmune factor in the “primed” spinal cord of PAE rats leading to susceptibility upon a second challenge. In support of this possibility, a mouse model PAE lead to significantly lowered brain microvascular glucose transporter (GLUT-1) expression, a marker of functional brain microvessels [58]. BIRT-377 significantly decreases LFA-1 in PAE rats to levels similar to those measured from non-PAE Sac controls (Fig. 4), suggesting that “resetting” LFA-1 to basal levels may be sufficient to ultimately control PAE-induced sensitivity to allodynia.

### Alteration of normal SGC and cytokine responses is seen in DRGs of PAE rats following chronic minor CCI

**Fig. 5:**
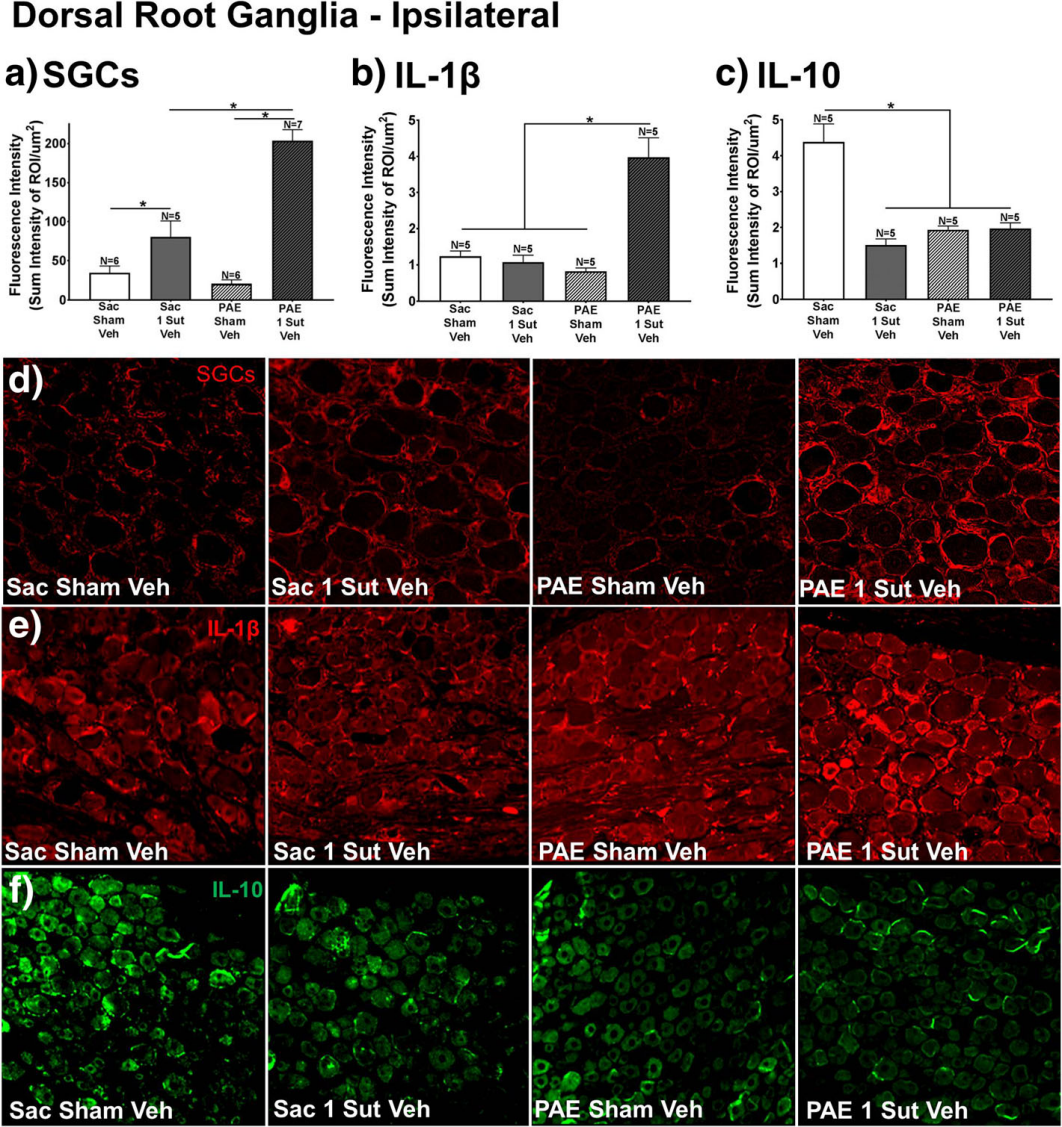
PAE results in elevated SGC activation and cytokine immunoreactivity in the dorsal root ganglia (DRG). Immunoreactivity of (**a**) SGCs (as measured by GFAP immunoreactivity), (**b**) IL-1β, and (**c**) IL-10 are determined for the ipsilateral L4-L6 DRG of PAE and Sac minor CCI vehicle injected rats at Day 4 post-injection (Day 32 after minor CCI) (rats from data shown in Fig. 1). (**a**) SGCs activation is significantly upregulated following minor CCI in both Sac and PAE rats. Additionally, compared to Sac rats, PAE rats have heightened SGCs activation. **b** Significant increases are observed following minor CCI in vehicle injected PAE rats compared to Sac rats with sham or minor CCI. **c** Sac-sham rats with vehicle display significantly greater IL-10 immunoreactivity compared to PAE-sham rats with vehicle rats. N = 5 rats per group. Asterisks indicate *p* < 0.05. **d** - **f** Representative images of SGCs, IL-1β, and IL-10 immunoreactivity used in IHC analysis are shown for Sac/ Sham/Veh, Sac/1-Sut/Veh, PAE/Sham/Veh, PAE/1-Sut/Veh, at 20x. “1-sut CCI” = “minor CCI”. N = 5 = 7 rats per group. Asterisks indicate *p* < 0.05

It is possible that L4-L6 DRGs of damaged sciatic nerve axons may respond to damage signals in an exaggerated manner, contributing to elevated glial and IL-1β occurring in the spinal cord. GFAP immunoreactivity for satellite glial cells (SGCs), IL-1β, and IL-10 in the L4-L6 DRGs from behaviorally verified rats (Day 32; rats from data shown in Fig. 1) is analyzed. The goal of this experiment is to identify neuroimmune factors influenced by PAE that may underlie susceptibility. Therefore, rats treated with BIRT-377 were intentionally omitted from this analysis. Statistical analysis demonstrate a main effect of PAE (SGCs, F_1,20_ = 17.74, *p* = 0.0004; IL-1β, F_1,16_ = 17.16, *p* < 0.0008; IL-10, F_1,16_ = 12.58, *p* = 0.0027), surgery (SGCs, F_1,20_ = 77.53, *p* < 0.0001; IL-1β, F_1,16_ = 24.87, *p* < 0.0001; IL-10, F_1,16_ = 25.7, *p* = 0.0001), and an interaction of PAE and surgery is seen (SGCs, F_1,20_ = 27.81, *p* < 0.0001; IL-1β, F_1,16_ = 30.67, *p* < 0.001; IL-10, F_1,16_ = 26.81, *p* < 0.001). Large and significant increases in the expression of GFAP in PAE rats with minor CCI compared to Sac controls with minor CCI are observed (Fig. 5a), suggesting that SCGs from PAE are more reactive/activated only following an injury, thereby unmasking satellite glial cell sensitization. The data show significant increases in IL-1β only in PAE rats following minor CCI (Fig. 5b). Interestingly, compared to Sac-sham rats displaying basal levels of IL-10 immunoreactivity, Sac rats with minor CCI, PAE-sham rats and PAE rats with minor CCI all display dramatically reduced basal levels of IL-10 immunoreactivity (Fig. 5c). Curiously, following minor CCI in Sac control rats, a reduction in IL-10 expression is observed despite a lack of allodynia (Fig. 5c), which is in contrast to the upregulation observed in the spinal cord (Fig. 3b). Interestingly, diminished IL-10 expression in the DRG following a 28-day CCI has previously been demonstrated in the DRG [42, 61], albeit the current CCI is a dramatically diminished injury. It is possible that the lack of elevated IL-10 in the Sac rats with minor injury may be a result of the significant reduction of damage applied to the sciatic nerve in the current report, as the diminished injury may be insufficient to elicit a modest “normal” compensatory IL-10 response at the peripheral nerve. Representative images (Fig. 5d-f) of SGCs, IL-1β, and IL-10 are shown.

### Spinal IL-1β is necessary for induction of a 10-day allodynia in PAE rats

**Fig. 6:**
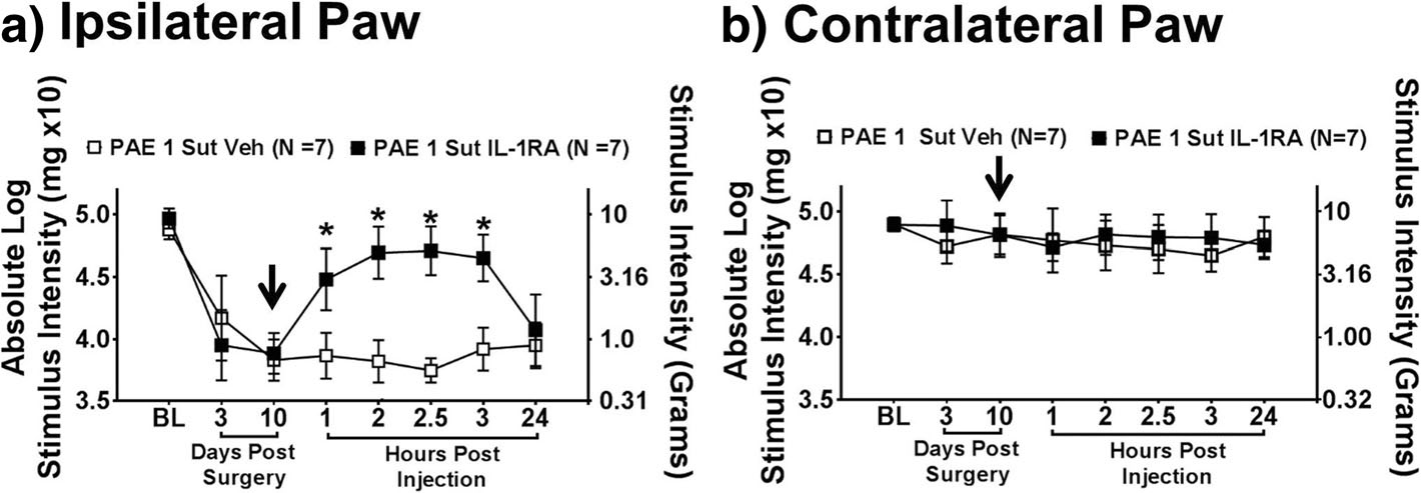
Spinal IL-1β is necessary for PAE induced allodynia following minor CCI. A separate group of PAE rats were used to determine whether IL-1β is necessary for induction of allodynia following minor CCI. PAE rats with minor CCI receive either i.t. IL-1 receptor antagonist (IL-1RA) or vehicle and (panel **a**) ipsilateral and (panel **b**) contralateral hindpaw responses are assessed. Prior to surgery, no hindpaw response threshold differences are observed between vehicle and IL-1RA groups. Following minor CCI and prior to injection, similar levels of robust ipsilateral allodynia is observed in both groups, while contralateral hindpaw responses remain unchanged. PAE rats receiving IL-1RA reveal ipsilateral reversal from allodynia by 1 h that persisted throughout the 3 h time-course compared to vehicle injected rats. By 24 h post-injection, all rats demonstrate full ipsilateral allodynia, with no significant differences observed between groups. “1-sut CCI” = “minor CCI”. *N* = 7 rats per group. Asterisks indicate *p* < 0.05

**Fig. 7:**
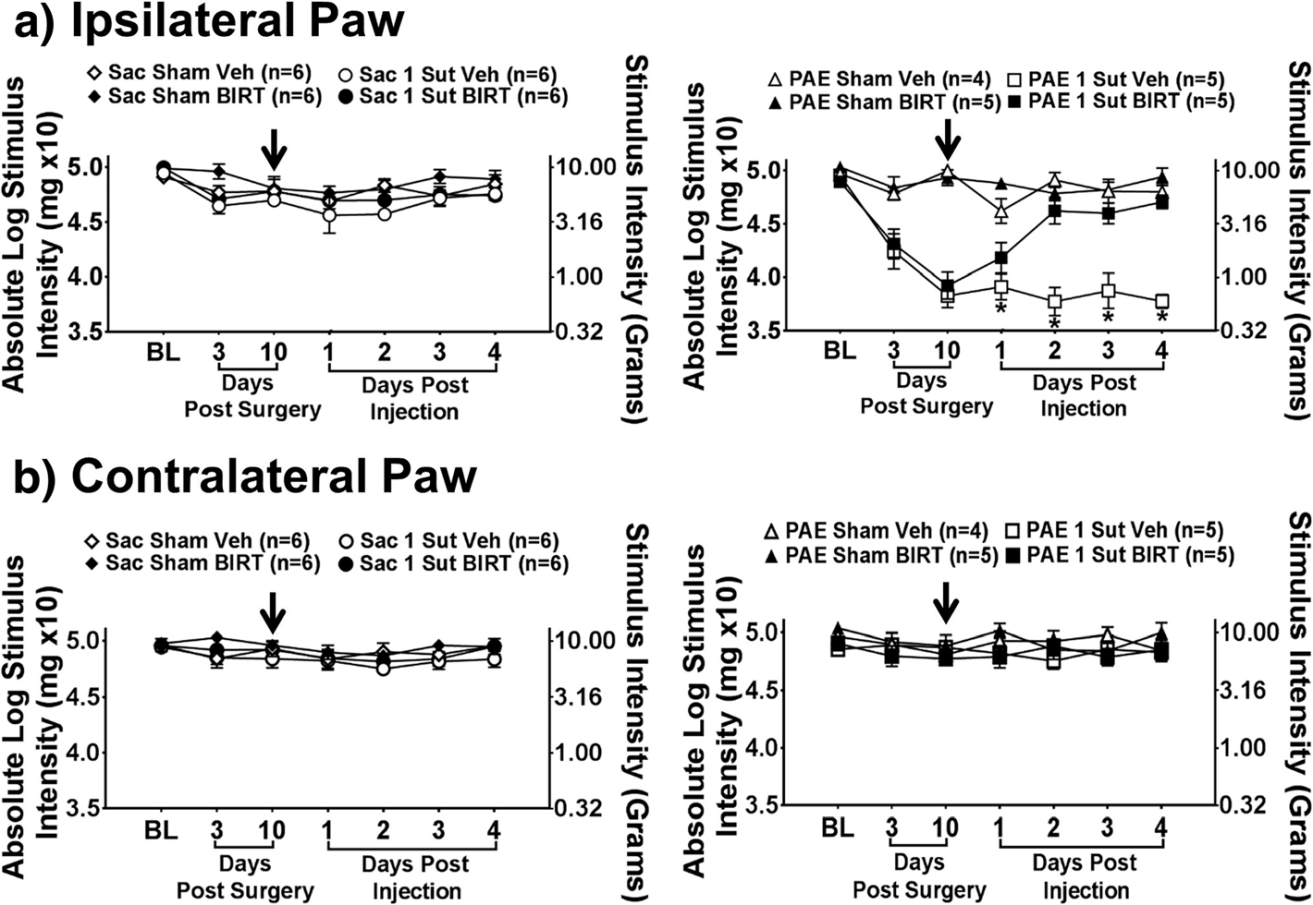
BIRT-377 reverses PAE induced allodynia following i.t. injection. **a-b** At baseline (BL), rats displayed no differences in hindpaw sensitivity. Additionally, sham groups maintain normal sensitivity throughout the time-course demonstrating that repeated von Frey behavioral testing has no significant effects on touch stimuli. Additionally, PAE, but not Sac rats, develop robust unilateral allodynia as measured on Day 3 and 10. Following injection of BIRT-377, PAE rats return to normal sensitivity through Day 4 post-injection at which time, the experiment was terminated for tissue collection. “1-sut CCI” = “minor CCI”. Arrow indicates when i.t. injection was given. N = 4–6 rats per group. Asterisks indicate *p* < 0.05

Several prior reports indicate the action of spinal IL-1β is necessary for the early and chronic phase of allodynia induced by the standard CCI model [36, 38]. The current report reveals that following a minor 32-day CCI, both Sac and PAE rats display elevated spinal IL-1β despite the absence of allodynia in Sac-minor CCI rats (Fig. 3a). leaving the role of spinal IL-1β ambiguous in PAE allodynia. The goal of the current experiment was to assess whether spinal IL-1β is necessary for a 10-day allodynia in PAE rats induced by minor peripheral nerve injury. Pharmacological intervention of spinal IL-1β with intrathecal (i.t.) IL-1 receptor antagonist (IL-1RA) was examined to determine if changes in PAE allodynia could be observed. To accomplish this, a separate group of PAE rats were assessed prior to surgery to determine BL responses, which demonstrated no significant differences between groups (Fig. 6a-b) (Ipsilateral, F_1,12_ = 4.523, *p* = 0.055; Contralateral, F_1,12_ = 0.014, *p* = 0.907). Following minor CCI, all PAE rats developed allodynia relative to their BL values (Fig. 6), replicating our prior observations (Figs. 1 and 7) (Ipsilateral, F_1,12_ = 0.743, *p* = 0.406; Contralateral, F_1,12_ = 2.213, *p* = 0.163). On Day 10 after minor CCI immediately following hindpaw threshold values, i.t. IL-1RA reversed allodynia almost to pretreated BL levels. Compared to vehicle, allodynia is reversed with hindpaw sensitivity initially changing within 1 h after the injection. This reversal is sustained for 3 h post-injection (Fig. 6) (Ipsilateral, F_1,12_ = 139.459, *p* < 0.001; Contralateral, F_1,12_ = 1.089, *p* = 0.317). Full allodynia returns by 24 h post-injection, replicating prior reports of the duration of action of IL-1RA (Ipsilateral, F_1,12_ = 0.887, *p* = 0.365; Contralateral, F_1,12_ = 0.743, *p* = 0.406) [14, 27, 52]. No significant differences are seen in sensitivity within the contralateral hindpaw throughout the behavioral time-course (Fig. 6b). This study establishes that in PAE rats with minor CCI, the susceptibility of developing allodynia is mediated, at least in part, by the actions of IL-1β in the spinal cord [45]. However, it remains unclear whether IL-1β acts in concert with other proinflammatory cytokines such as TNF-α that ultimately generates the observed allodynia. The role of spinal TNF-α has previously been shown to participate in the development of allodynia in a rat model of local spinal glial activation [37], and thus, may be an important consideration in PAE generating susceptibility to neuropathy.

### I.T. BIRT-377 reverses PAE induced allodynia at day 10 post-surgery

Prior reports and the initial studies detailed in the present report demonstrate robust unilateral allodynia through Day 28 [49] or Day 32 after minor CCI in PAE rats. To further explore the spinal mechanisms underlying early-established allodynia following minor injury, adult PAE and Sac rats are subjected to either sham or minor CCI surgery, and upon established allodynia observed on Day 10, the effect of i.t. BIRT-377 on allodynia was assessed. As before (Fig. 1), BL light touch sensory thresholds are similar between all groups (Fig. 7) (ipsilateral, F_1,35_ = 0.687, *p* = 0.413; contralateral, F_1,35_ = 0.041, *p* = 0.840). Furthermore, following CCI, a main effect of alcohol exposure (ipsilateral, F_1,35_ = 52.497, *p* < 0.0001; contralateral, F_1,35_ = 1.929, *p* = 0.174), surgery (ipsilateral, F_1,35_ = 148.009, *p* < 0.0001; contralateral, F_1,35_ = 3.118, *p* = 0.086) and an interaction between alcohol exposure and surgery (ipsilateral, F_1,35_ = 87.448, *p* < 0.0001; contralateral, F_1,35_ = 0.387, *p* = 0.538) is seen only in the ipsilateral hindpaw. Additionally, while normal sensitivity throughout the timecourse is maintained near BL thresholds in Sac groups with sham or minor CCI, a pronounced unilateral allodynia is observed ipsilateral to minor sciatic CCI in PAE rats while no change in contralateral hindpaw sensitivity is observed (Fig. 7a-b) (ipsilateral, F_1,35_ = 89.118, *p* < 0.0001; contralateral, F_1,35_ = 0.710, *p* = 0.405). Following i.t. BIRT-377 or vehicle injection in Sac rats, hindpaw thresholds remain stable throughout the 4-day timecourse, hovering close to pretreatment BL values. However, i.t. BIRT-377 induces complete reversal of ipsilateral allodynia for the remainder of the timecourse while contralateral hindpaw thresholds continue to remain stable and non-allodynic (Fig. 7a-b) (ipsilateral, F_1,35_ = 9.808, *p* = 0.003; contralateral, F_1,35_ = 0.032, *p* = 0.858). These data demonstrate that LFA-1 blockade during early-established (10-day) allodynia may alter factors similar to those driving enduring (Day 28 or longer) allodynia. Following the completion of behavioral verification of i.t. BIRT-377 efficacy on Day 10, sciatic nerve, DRG and lumbar spinal cord were dissected and candidate protein and mRNA for cytokines and chemokines were examined, as detailed below.

### PAE may induce allodynia by altering proinflammatory factors at the sciatic nerve

To assess whether BIRT-377 changes the spinal proinflammatory environment resulting in the reversal of allodynia in PAE rats, ipsilateral sciatic nerve (SCN) as well as ipsilateral and contralateral (as a potential within animal biochemical control) lumbar spinal cord were collected at Day 4 post-injection (Day 14; rats from data shown in Fig. 7) at maximal i.t. BIRT-377 efficacy to examine protein using the V-plex immunoassay. Expression levels of IL-10, IL-1β, and TNFα were measured in the SCN and the ipsilateral L4-L6 dorsal lumbar spinal cord as well as CXCL1 in the SCN. Results for the SCN demonstrate that following vehicle injection, minor CCI induces a modest but significant increase in IL-10 expression in PAE rats compared to Sac rats (F_7,35_ = 1.672, *p* = 0.0271). These results are in stark contrast to our prior report demonstrating that protein IL-10 levels are dramatically blunted in PAE rats with CCI [42]. In the prior report, the standard CCI model was applied and the sciatic nerves from Sac-CCI rats revealed a 5-fold increase in IL-10 protein over PAE rats with standard CCI, with one distinction being that tissues were collected at Day 28 after CCI [42]. It is also possible that in the current study, minor CCI in Sac rats is insufficient to stimulate peri-sciatic immune cells to produce a significant IL-10 protein response. As such, IL-10 responses to minor CCI in Sac animals cannot unmask the IL-10 response-deficit to injury in PAE rats.

**Fig. 8:**
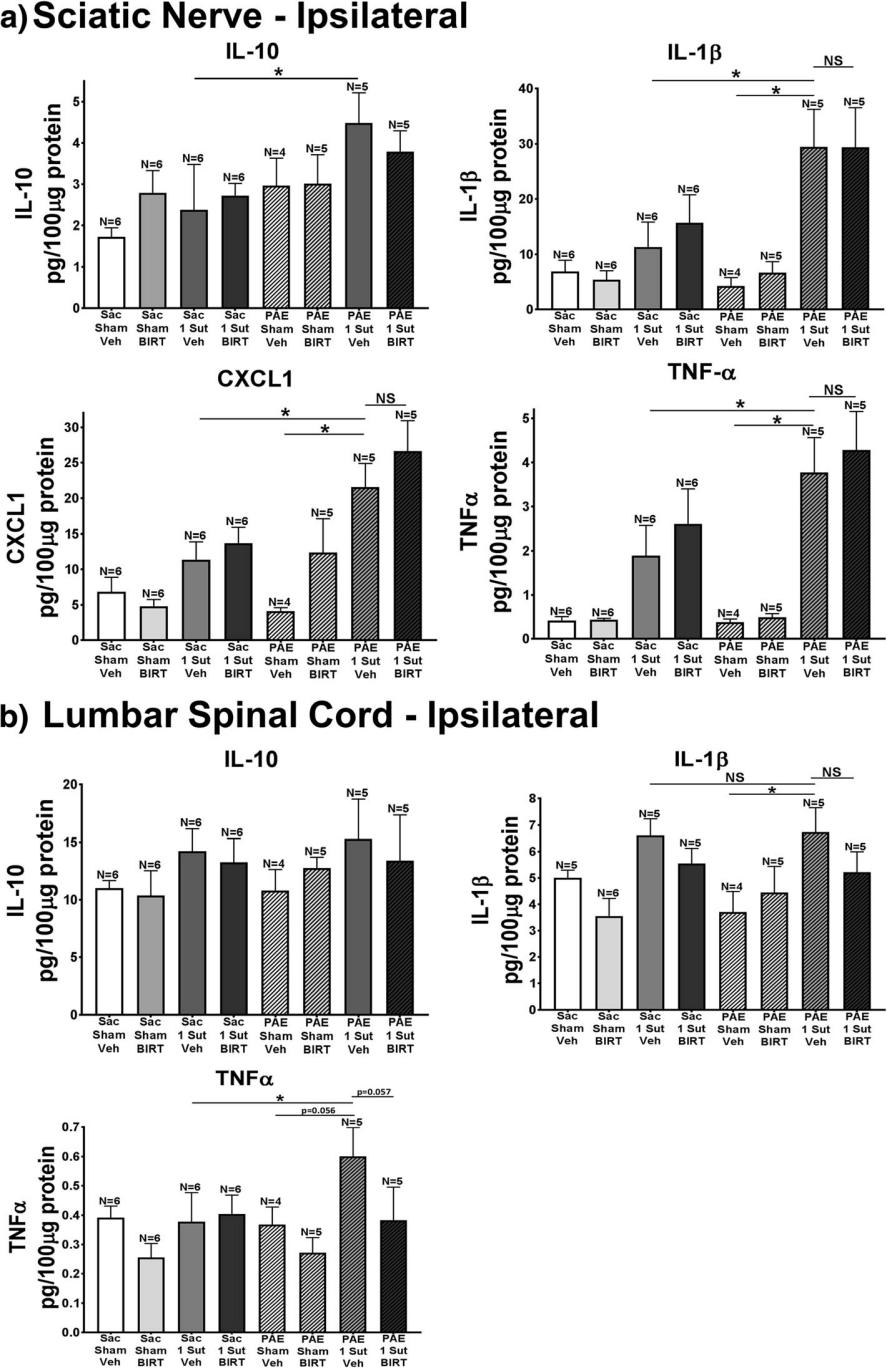
PAE alters cytokine expression in the ipsilateral sciatic nerve and spinal cord. Ipsilateral sciatic nerve and spinal cord are collected at Day 4 post-injection (Day 14 after minor CCI) (rats from data shown in Fig. 7) and examined by multiplex protein analysis for levels of IL-10, IL-1β, TNFα, and CXCL1 (sciatic nerve only). Levels of protein are expressed as picograms of target protein per 100 μg of total protein. Data for ipsilateral (**a**) sciatic nerve and (**b**) lumbar spinal cord are represented. **a** IL-10 significantly increased in PAE rats with minor CCI given vehicle injection compared to Sac CCI vehicle injected rats. Additionally, compared to Sac/minor CCI vehicle injected rats and PAE sham vehicle rats, PAE minor CCI rats with vehicle injection displayed elevated levels of IL-1β, TNFα, and CXCL1. **b** IL-10 expression did not change significantly throughout any groups in the ipsilateral lumbar spinal cord. PAE minor CCI vehicle injected rats had significant increases in IL-1β expression compared to PAE sham vehicle rats. Additionally, no significant differences are seen in IL-1β between Sac minor CCI vehicle rats and PAE minor CCI vehicle rats. Significant differences in TNFα are seen between Sac minor CCI vehicle rats and PAE minor CCI rats. “1-sut CCI” = “minor CCI”. N = 4–6 rats per group. Asterisks indicate *p* < 0.05

Despite the ambiguous results of IL-10 protein from sciatic nerve tissues, significant and reliable increases in protein IL-1β, CXCL1, and TNFα were observed following minor injury, with the magnitude of protein increases observed to be far greater in PAE rats with minor CCI (Fig. 8a) (IL-1β: F_7,35_ = 5.224, Sac minor CCI: *p* = 0.0060; PAE Sham Veh: *p* = 0.0008; CXCL1: F_7,35_ = 5.224, Sac minor CCI: *p* = 0.0158; PAE Sham Veh: *p* = 0.0004; TNFα: F_7,35_ = 5.224, Sac minor CCI: *p* = 0.0239; PAE Sham Veh: *p* = 0.0005). No significant differences were seen between PAE minor CCI vehicle groups and PAE minor CCI BIRT groups (F_7,35_ = 5.224, IL-10: *p* = 0.4716; IL-1β: *p* = 0.9904, TNFα: *p* = 0.5436, CXCL1: *p* = 0.2309). Ipsilateral lumbar spinal cord IL-10 data reveal no significant differences between groups.

As observed with protein collected from sciatic nerves, IL-10 expression in the spinal cord did not show blunted IL-10 responses to injury in PAE rats compared to Sac rats. These observations were surprising, as we predicted that the lack of allodynia observed in Sac rats with minor CCI was due to elevated spinal IL-10 at this early 10-day timepoint of chronic allodynia. Interestingly, elevated spinal IL-10 is observed in Sac rats with a 32-Day allodynia (Figs. 3b and 5c), suggesting that during the early spinal response to peripheral injury, significant changes in IL-10 protein levels is not requisite for controlling the development of neuropathy from a minor injury. Analysis of IL-1β expression demonstrated significant increases in PAE minor CCI vehicle injected rats compared to PAE sham vehicle rats (F_7,32_ = 5.224, Sac minor CCI: *p* = 0.0097). Also at this early timepoint, similar IL-1β protein responses are observed in both PAE and Sac rats with minor CCI and vehicle injection despite ongoing allodynia only observed in PAE rats (F_7,32_ = 5.224, Sac minor CCI: *p* = 0.9070). Taken together, these data suggest other neuroimmune factors may play a role in generating allodynic susceptibility either by synergizing with IL-1β or by acting downstream of IL-1β. Moreover, while a modest reduction of IL-1β in allodynic PAE rats treated with BIRT-377 is measured, the reduction is not significant.

Spinal TNFα is significantly increased in PAE allodynic rats compared to Sac non-allodynic rats with minor CCI (F_7,32_ = 5.224, Sac minor CCI: *p* = 0.0432). Following BIRT-377 treatment, a strong trend towards reduced levels of TNFα is observed in pain-reversed PAE rats (Fig. 8b) (*p* = 0.057). Lumbar spinal cord contralateral to the sciatic CCI did not show significant differences between rat groups (data not shown), which coincides with unaltered contralateral hindpaw threshold levels. As eluded to above, these data demonstrate that pronounced peri-sciatic immune reactivity may drive other neuroimmune factors in the spinal cord that synergize with IL-1β resulting in allodynia in PAE rats following a minor CCI. Indeed, IL-1β and TNFα have been demonstrated to act synergistically [34] and speculated to underlie pathological pain from heightened spinal glial activation [37]. It is clear, however, that BIRT-377 reverses early-phase PAE allodynia by altering the action of multiple cytokines.

### Minor CCI alters CCL2 and CXCL1 in the ipsilateral SCN and DRG

**Fig. 9:**
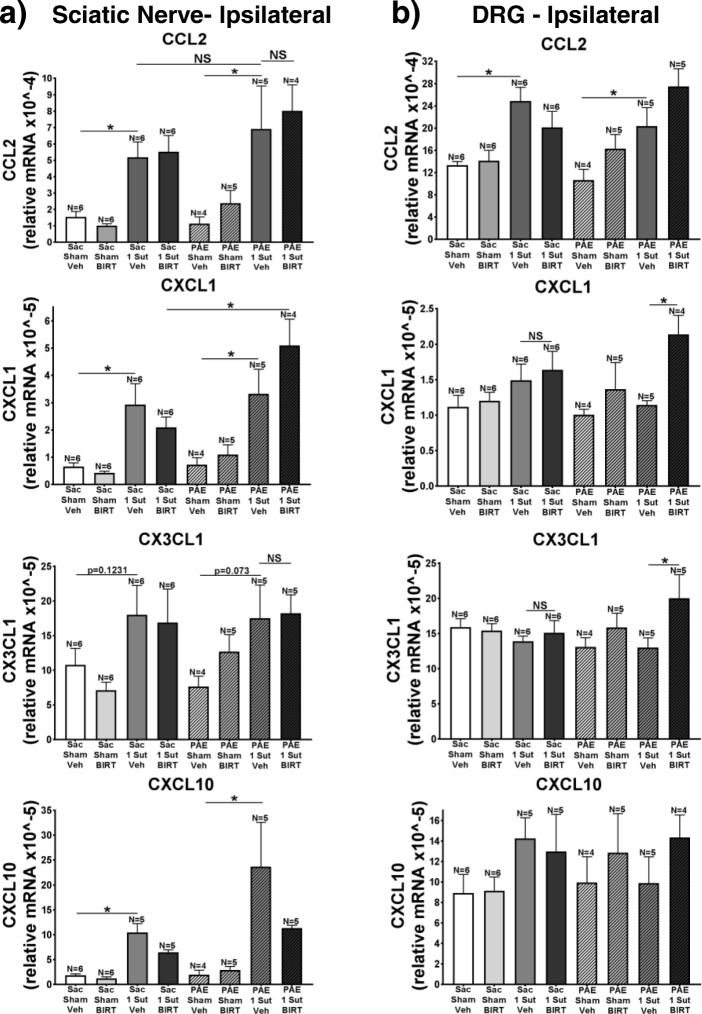
I.t. BIRT-377 alters mRNA expression of specific chemokines only in PAE neuropathic rats. Ipsilateral SCN and DRG are collected from rats at Day 4 post-injection (Day 14 after minor CCI) for qRT-PCR analysis (rats from data shown in Fig. 7). Messenger RNA expression from ipsilateral (**a**) In SCN, CCL2, CXCL1, CXCL10 are significantly increased following minor CCI in PAE rats compared to Sac rats. CXCL1 is further increased in PAE rats vs. Sac rats given BIRT377. (**b**) In DRG, CCL2 is increased in Sac and PAE rats with minor injury while treatment with BIRT377 results in CXCL1 and CX3CL1 increases in PAE rats with minor injury

**Fig. 10:**
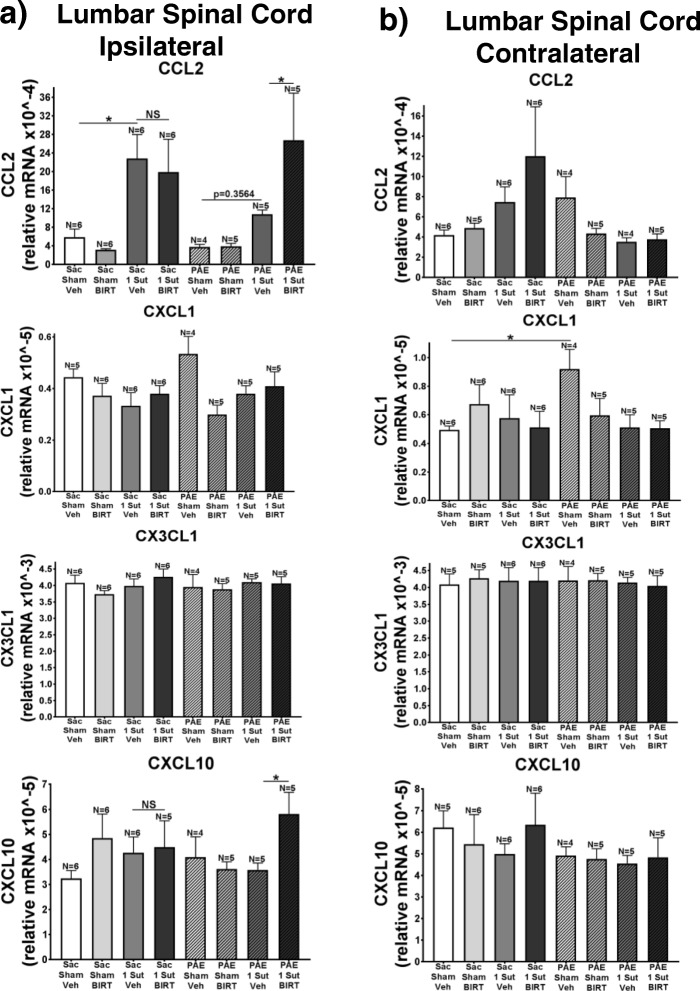
(**a**) ipsilateral and (**b**) contralateral lumbar spinal cord are shown. (**a**) The expression of CCL2 is increased following minor CCI in Sac and PAE rats. Additionally, BIRT-377 increased CCL2 and CXCL10 only in PAE rats with minor CCI. (**b**) Contralateral dorsal spinal cord revealed significant CXCL1 increases from PAE compared to Sac Sham rats. “1-sut CCI” = “minor CCI”. N = 4–6 rats per group. Asterisks indicate *p* < 0.05

Previous reports demonstrate that following sciatic nerve injury, the chemokines CCL2, CXCL1, CX3CL1, and CXCL10 become upregulated [2, 21, 29, 64]. Furthermore, separate studies show that astrocytes may mediate allodynia through the release of CXCL1 [9], CX3CL1, and CXCL10 [30]. Interestingly, minor CCI results in the activation of spinal astrocytes but not microglia in aged rats [49]. In addition, PAE alters the release of CCL2 [42]. Together, these reports suggest that susceptibility to allodynia in PAE rats may result from alterations in spinal cord chemokine production and release due to primed spinal glial activation. Thus, in order to characterize the chemokine profile following minor CCI, qRT-PCR is used to assess the gene activation of CCL2, CXCL1, CX3CL1, and CXCL10 in the ipsilateral SCN (Fig. 9a), DRG (Fig. 9b), and ipsilateral (Fig. 10a) and contralateral lumbar spinal cord (Fig. 10b) (Day 14; tissue collected from behaviorally verified rats from Fig. 7). SCN data show that only following CCI are significant increases seen in expression of CCL2, CXCL1, and CXCL10 in both Sac and PAE rats (Fig. 9a) (Sac: CCL2 F_7,34_ = 5.32, *p* = 0.0242, CXCL1 F_7,34_ = 5.32, *p* = 0.0033, CXCL10 F_7,34_ = 5.32, *p* = 0.0117; PAE: CCL2 F_7,34_ = 5.32, *p* = 0.0027, CXCL1 F_7,34_ = 5.32, *p* = 0.0038, CXCL10 F_7,34_ = 5.32, *p* = 0.0133). Interestingly, significant differences in CXCL1 from SCN are seen between PAE and Sac rats with CCI that received BIRT-377 (Fig. 9a) (F_7,34_ = 5.32, *p* = 0.0007). Assessment of the DRG demonstrated that CCL2 is upregulated following CCI in both Sac and PAE rats (Fig. 9b) (Sac: F_7,35_ = 5.26, *p* = 0.0014; PAE: F_7,35_ = 5.26, *p* = 0.0168). Only PAE rats with BIRT-377 display significant increases in CXCL1 and CX3CL1 (Fig. 9b) (CXCL1: F_7,34_ = 2.377, *p* = 0.0057; CX3CL1: F_7,35_ = 1.593, *p* = 0.0079). Overall, data suggest that minor CCI upregulates pain relevant chemokines from peri-sciatic immune cells, as one would predict, but that the magnitude of the immune cell response is greater in PAE rats.. Gene activation, as measured by mRNA analysis, reveals that immune signals known to drive leukocyte trafficking at the SCN and DRG during chronic neuropathy are similar between rats exposed to Sac or PAE.

### I.T. BIRT-377 alters chemokine expression only in PAE rats with minor CCI within the spinal cord

Assessment of mRNA within the ipsilateral and contralateral spinal cord reveals that minor CCI increases CCL2 gene activation in Sac rats compared to sham conditions (Fig. 10a) irrespective of BIRT-377 (Sac: F_7,35_ = 3.879, *p* = 0.0139). Interestingly, CCL2 gene activation is blunted in PAE rats with CCI in that no significant differences seen when compared to Sham rats (Fig. 10b). Significant differences in CCL2 and CXCL10 gene expression are only observed in PAE rats with minor CCI that received BIRT-377 (Fig. 10a) (CCL2: F_7,35_ = 3.879, *p* = 0.0321; CXCL10: F_7,35_ = 3.879, *p* = 0.0373). It should be noted that these increases are not significantly different when compared to Sac rats with minor CCI and BIRT-377 injection (Fig. 10a). Contralateral data demonstrated no significant differences in CCL2, CXCL1, and CXCL10 gene activation are seen between groups, which correspond with normal and stable sensitivity observed in the contralateral hindpaw (Figs. 1c-d and 7b) (F_7,35_ = 1.289, *p* = 0.0182). Interestingly, PAE alone was sufficient to induce increases in CXCL1 gene expression (Fig. 10b). The lack of changes in chemokines assessed in this report at the lumbar spinal cord may suggest that BIRT-377 may not work primarily by transcriptional regulation of these chemokines.

## Discussion

A growing body of evidence demonstrates that PAE leads to heightened immune reactivity peripherally, as well as in the brain and spinal cord [42, 49, 57]. Interestingly, the effects of PAE are not unmasked until after a subsequent insult has occurred [42, 49]. In addition to heightened immune reactivity, PAE alters the β2-adhesion molecule LFA-1, which is classically known to facilitate immune cell trafficking in response to CCL2-CCR2 signaling co-localized on same immune cells followed by migration to regions where tissue damage has occurred. In the present report, we identify a possible novel additional role of LFA-1 activation in that the local milieu is biased toward the proinflammatory cytokine network leading to susceptibility to pathological pain [42]. This study demonstrates that a minor injury results in enduring unilateral allodynia up to 32 days post-surgery only in PAE rats, in support of prior work [42, 49]. Furthermore, the current report and previous work demonstrate that PAE alone alters LFA-1, while separate reports show the negative consequences of LFA-1 on proinflammatory actions are influenced by IL-10 expression [12]. The current report supports that antagonism of LFA-1 suppresses allodynia. I.t. injection of the LFA-1 antagonist BIRT-377, abolishes a 28-Day enduring allodynia with maximum reversal occurring at Day 4 post-injection (Fig. 1). It is important to note this is a small molecule antagonist with high specificity to LFA-1, and it is not an antibody that could have unintended off-target effects. The present report additionally demonstrates that spinal and DRG glial activation as well as proinflammatory cytokine expression is reduced from an i.t. application of BIRT-377 (Figs. 2, 3 and 5). Together, these data are the first demonstration that LFA-1 is a potential therapeutic target to control primed neuroimmune responses leading to neuropathic pain.

The pain suppressive effects of BIRT-377 may also act to prevent further spinal infiltrating peripheral leukocytes, given peripheral leukocytes express LFA-1 [5, 53]. Although speculative, it is possible that following CCI, peripheral leukocytes migrate into the spinal cord where the presence of BIRT-377 may result in these cells acquiring an anti-inflammatory phenotype. Thus, these cells may aid in the reversal of allodynia through release of their own IL-10. LFA-1 is expressed by peripheral leukocytes which have been demonstrated to aid in the induction of allodynia both at the site of injury as well as in the spinal cord [1, 8, 43, 53]. Future studies need to be done examining the potential effects of intravenous (i.v.) BIRT-377 in suppressing CCI-induced allodynia to determine the role that peripheral leukocytes within the spinal cord exert in the induction and reversal of allodynia in PAE rats with minor injury. Given Lifitegrast, an LFA-1 antagonist, has been approved by the US Food and Drug Administration for the treatment of dry eye [1], future examination of BIRT-377 as a clinically approved pain therapeutic could be achievable in the near future.

The actions of spinal IL-1β as a critical mediator of PAE-induced early-onset allodynia was supported in the current report, as blocking IL-1β with i.t. ILRA on Day 10 of minor CCI-induced allodynia transiently reversed allodynia in PAE rats (Fig. 6). This suggests that spinal IL-1β action is necessary in part to induce allodynia following minor injury, but may not be the only factor critical in establishing early-onset allodynia. One possible candidate factor, a proinflammatory cytokine that is capable of enhancing IL-1β actions, is TNFα. IL-1β and TNFα have been demonstrated to act synergistically [34]. Furthermore, the significant elevation of TNFα protein in the dorsal horn of the spinal cord from PAE-CCI rats with a 10-day early onset allodynia suggests that PAE may shift sensitivity toward a TNFα bias that sets into action a series of proinflammatory neuroimmune consequences. In support, a prior study demonstrated that TNFα triggers a cytokines cascade including IL-1β yielding adverse neuronal function [56], and it has been established that primed astrocytes in the degenerating brain produce exaggerated TNFα, IL-1β CCL2 protein [19]. These data suggest that increased sensitivity to TNFα, in particular by astrocytes, is present in the PAE CNS, a concept recently supported by a study demonstrating that astrocytic connexin 43 (Cx43) in a mouse model of PAE is significantly upregulated that could underlie PAE-induced cerebral hyperexcitability [44]. Elevated Cx43 may reflect destabilized astrocytic responses to glutamate neurotransmission, as a separate report extended the role of basal Cx43 on astrocytes as a buffer for extracellular glutamate, which under neuropathic conditions or application of TNFα, Cx43 expression becomes significantly downregulated [40, 44]. Thus, while speculative, the astrocyte sensitivity to TNFα may be increased that synergizes with IL-1β in PAE offspring upon induction/early establishment of minor injury.

In spinal cord collected from Day 32 post-surgery, IHC data show compensatory upregulation of IL-10 following minor CCI in Sac control rats. In contrast, PAE rats with minor CCI demonstrate blunted IL-10 responses (Fig. 3b), supporting the notion that PAE dysregulates IL-10 compensatory responses under conditions of enduring allodynia. DRG data demonstrate a downregulation of IL-10 following minor CCI in Sac controls but blunted IL-10 responses as a result of PAE alone (Fig. 5c), while Day 14 post-surgery rats show increased protein IL-10 at the SCN only in PAE rats with minor CCI (Fig. 8). These differences could be attributed to multiple factors including the distinct cell types capable of generating present in the DRG (satellite glia), SCN (peripheral immune cells), and spinal cord (astrocytes, microglia and infiltrating immune cells) that are programmed to respond to tissue injury while minimizing unintended bystander damage. The timepoints at which the tissue was analyzed; that is Day 14 (Fig. 1) or Day 32 (Fig. 7) post-surgery, which may be an additional critical factor, as immune and glial cell phenotypes adapt with the duration of exposure to the tissue environment. Previous reports demonstrate that at Day 10 CCI, IL-10 responses decrease both in the dorsal horn spinal cord and DRG in rats (without PAE manipulation) [61]. This finding is replicated in the current report of Day 32 DRG data (Fig. 5c) but not from spinal cord (Fig. 3b). Additionally, Noor and colleagues demonstrate increases in IL-10 expression following CCI in Sac control rats, albeit with application of a standard CCI model at Day 28, but a blunted IL-10 response in PAE rats [42]. These data support the notion that IL-10 expression is different based on the time point at which analysis occurs. Thus, it is possible that the IL-10 expression changes over time following CCI surgery. A final potential factor that could influence differences in protein levels collected at different timepoints could be the detection method used. For example, IHC semi-quantitative analysis (in Figs. 3 and 5) versus protein assay by electrochemiluminescence (Fig. 8). One of the benefits to using IHC to assess protein expression is the ability to quantify distinct anatomical regions of the DRG and the spinal cord. This allows for careful characterization within specific regions that sensory neurons from the DRG project to within the spinal cord. Discrete anatomical analysis is not possible when using electrochemiluminescence to analyze protein because this method requires whole tissue homogenization which includes grey and white matter thereby obscuring accurate protein signal preventing us from determining discrete protein levels within discrete anatomical regions.

The current data show that although microglia become upregulated following minor injury, astrocytes show greater activation in PAE rats when compared to controls (Fig. 2). Previous studies show that LFA-1 is upregulated on spinal microglia (and possibly macrophages) as a result of PAE alone [42], and interestingly, previous reports show that LFA-1 is expressed on microglia whereas its ligand ICAM-1 is expressed on astrocytes [3, 28, 48]. In light of these prior reports, it is possible that during pathological conditions, microglia may communicate to astrocytes expressing ICAM-1 resulting in an ICAM-1 to LFA-1 interaction consequently inducing proinflammatory cytokine and chemokine production and signaling during the induction of allodynia. Furthermore, in addition to the current data reported here (Fig. 2a), prior reports show that PAE is able to alter astrocytes within the spinal cord following peripheral nerve injury [42, 49]. Together these data suggest that although spinal microglia are activated similarly between Sac controls and PAE, dysfunctional astrocytes in PAE rats may be the key players, and through their sensitization, may require only modest stimulation to undergo augmented activation. This alteration could reasonably underlie the induction of allodynia following minor injury.

The current study demonstrates a key role for IL-1β following minor CCI (Fig. 3a) and is necessary for the maintenance of allodynia in PAE rats (Fig. 6). The current report also supports the possible involvement of other complimentary cytokines such as TNFα, in which it has been shown to be upregulated by IL-1β [6], can act synergistically with IL-1β during neuropathy [23] and is involved in the induction and maintenance of allodynia [15, 59]. Furthermore, previous reports show that TNFα is able to induce the expression of LFA-1 [20]. As noted above, LFA-1 activation on immune cells is impacted by IL-10 [12, 25]. Thus, TNFα may play a role in the susceptibility to allodynia created by PAE following minor injury, in part, by its actions of increasing spinal LFA-1 and consequent immune and glial sensitization. Overall, the data support the possibility that the actions of both IL-1β and TNFα may mediate the induction of allodynia in PAE rats with minor CCI.

Interestingly, the activation marker, GFAP in satellite glial cells (SGCs) within the DRG is upregulated as a result of minor injury only in PAE rats (Fig. 5a). These cells typically provide sensory neuron cell bodies with essential amino acids as well as clearing toxic substances from the extracellular space [18, 39, 55, 62]. During pathological conditions, pain signals from the periphery travel into the DRG and on to the dorsal horn spinal cord [39]. In the DRG, SGCs can communicate with neurons through the release of IL-1β and IL-10 [55]. The current data demonstrate that following minor injury, IL-1β immunoreactivity is significantly increased whereas IL-10 expression appears blunted within the DRG as a result of PAE alone (Fig. 5), suggesting that pro- and anti-inflammatory SGC responses to minor challenges are dysregulated as a result of PAE. The data demonstrate that one source of heightened IL-1β expression and dysregulated IL-10 expression occurs in the DRG. Ultimately, unchecked IL-1β actions in the DRG due to the lack of IL-10 can contribute to amplified pain signals projecting into the spinal cord during minor CCI.

Curiously, microglial states of activation are not always altered by PAE, particularly under models of moderate PAE [62]. In support of these prior reports, data from the current study also show that in spinal cords of PAE rats with minor injury, a significant upregulation of the astrocyte activation marker, GFAP is present but no differences in microglial activation are observed in PAE neuropathic and non-neuropathic Sac rats. In fact, levels of Iba1 immunoreactivity (a potential indicator of heightened microglial activation) was similar between Sac rats with minor injury (no allodynia) and PAE rats with minor injury (with allodynia) (Fig. 2). These data suggest that PAE induced sensitivity to allodynia may be mediated through astrocyte mechanisms. Interestingly, prior reports show that PAE downregulates the expression of the astrocyte specific glutamate transporter GLAST that coincides with a decrease in glutamate uptake [7]. In addition, a separate report demonstrates that only after GLAST function is inhibited in addition to the other glutamate transporters, GLT-1 (on glia and neurons) and EAAC1 (solely on neurons), potentiated allodynia is observed [54]. Curiously, it is demonstrated that following a standard (4-suture) sciatic CCI, potentiated allodynia is observed in PAE rats [42]. Together, these data suggest that PAE may lead to susceptibility to allodynia by altering the sensitivity of astrocytes, and in doing so, also alter the functional response of the astrocyte-specific glutamate transporter GLAST upon minor challenge.

## Conclusions

In conclusion, data from the current report support the possibility that PAE may lead to susceptibility to developing pathological pain through the alteration of LFA-1 function and expression, and increased astrocyte sensitization to cytokines. By antagonizing the spinal activation of LFA-1, a switch in the local environment within the spinal cord from proinflammatory to an anti-inflammatory phenotype occurres, abolishing allodynia. Normal protective responses of astrocytes are diminished as a consequence of PAE, which is unmasked only following a second minor challenge in adulthood; the challenge being minor peripheral nerve injury. Additionally, these data demonstrate that PAE suppresses healthy IL-10 basal and compensatory expression, which may aid in the induction of a proinflammatory bias following injury and over-reactive astrocytes. Overall, these data shed light on potential mechanisms underlying PAE’s glial and neuroimmune actions that renders one vulnerable to chronic CNS diseases such as neuropathic pain.
